# A Transfer Function-Based Binary Version of Improved Pied Kingfisher Optimizer for Solving the Uncapacitated Facility Location Problem

**DOI:** 10.3390/biomimetics10080526

**Published:** 2025-08-12

**Authors:** Ayşe Beşkirli

**Affiliations:** Department of Medical Education and Informatics, Faculty of Medicine, Karamanoğlu Mehmetbey University, 70200 Karaman, Türkiye; aysebes@kmu.edu.tr

**Keywords:** pied kingfisher optimizer, transfer function, uncapacitated facility location problem, binary optimization

## Abstract

In this study, the pied kingfisher optimizer (PKO) algorithm is adapted to the uncapacitated facility location problem (UFLP), and its performance is evaluated. The PKO algorithm is binarized with fourteen different transfer functions (TF), and each variant is tested on a total of fifteen different Cap problems. In addition, performance improvement was realized by adding the Levy flight strategy to BinPKO, and this improved method was named BinIPKO. The experimental results show that the TF1 transfer function for BinIPKO performs very well on all problems in terms of both best and mean solution values. The TF2 transfer function performed efficiently on most Cap problems, ranking second only to TF1. Although the other transfer functions provided competitive solutions in some Cap problems, they lagged behind TF1 and TF2 in terms of overall performance. In addition, the performance of BinIPKO was also compared with the well-known PSO and GWO algorithms in the literature, as well as the recently proposed APO and EEFO algorithms, and it was found that BinIPKO performs well overall. In line with this information, it is seen that the IPKO algorithm, especially when used with the TF1 transfer function, provides an effective alternative for UFLP.

## 1. Introduction

Optimization is a process that aims to determine the optimal solution values of decision variables to minimize or maximize the objective function defined for a problem [1,2]. Optimization is a tool used to determine optimal solutions in many fields, from engineering to logistics planning. In real-world applications, problems such as 0–1 knapsack [3,4,5,6,7], feature selection [8,9,10,11,12], traveling salesman [13,14,15], wind turbine layout [16,17,18,19,20], and uncapacitated facility location [21,22,23] have a binary structure where the decision variables can only take the values 0 or 1. The high computational cost of traditional methods has recently increased the interest in nature-inspired metaheuristic algorithms for solving binary optimization problems [24]. In this direction, various metaheuristic algorithms have been converted into binary form and used to solve binary optimization problems. Among the studies conducted in this context, Baş and Ülker [25] converted the social spider algorithm (SSA) into binary form using four different transfer functions and proposed binary versions of SSA. Beşkirli and Dağ [26] proposed a binary variant of the Harris hawks algorithm called HHObin using eight different transfer functions. In order to test the performance of the proposed HHObin algorithm, they applied it to the wind turbine layout problem. Pan et al. [27] proposed a binary version of the fish migration optimization algorithm using transfer functions. Hu et al. [28] transformed the gray wolf algorithm into a binary form by using S-shaped and V-shaped transfer functions. They named the binary gray wolf algorithm BGWO. They applied their proposed BGWO algorithm to the feature selection problem to demonstrate its effectiveness. Mafarja et al. [29] proposed a binary version of the grasshopper optimization algorithm called BGOA using S-shaped and V-shaped transfer functions. They applied their proposed BGOA method to feature selection problems. Yıldızdan and Baş [7] binarized the artificial jellyfish search algorithm for solving the knapsack problem by using eight different transfer functions. Arora and Anand [30] proposed a binary variant of the butterfly optimization algorithm using transfer functions for feature selection. Beşkirli et al. [17] converted the artificial algae algorithm into binary form using transfer functions. Then, they applied the proposed binary method to the wind turbine placement problem. Ervural and Hakli [3] converted the reptile search algorithm to binary form using transfer functions for solving 0–1 knapsack problems. Büyüköz and Hakli [31], in their study, binarized the honey badger algorithm by using various transfer functions. They applied their proposed binary honey badger algorithm to 0–1 knapsack problems. Guo et al. [32] transformed the particle swarm optimization (PSO) algorithm into a binary structure using transfer functions. They compared the performance of their proposed binary PSO on a benchmark dataset. Varzaneh et al. [33] converted the horse swarm optimization algorithm into binary form using S-shaped and V-shaped transfer functions. They tested the efficiency of their proposed method on standard benchmark datasets. Then they applied their proposed binary method to the feature selection problem. Balakrishnan et al. [34] converted the African vulture optimization algorithm to binary by using S-shaped and V-shaped transfer functions. After testing the performance of their proposed method on a benchmark dataset, they applied it to the feature selection problem. Basset et al. [4] proposed a binary version of the balance optimization algorithm using S-shaped and V-shaped transfer functions. They then used their proposed binary balance optimization algorithm to solve the 0–1 knapsack problem.

In this study, the pied kingfisher optimizer (PKO) [35], a metaheuristic algorithm inspired by the hunting and feeding behavior of the pied kingfisher bird, is considered. The PKO algorithm, which works in the continuous search space, is adapted to the binary optimization problem using fourteen different transfer functions (TF) in this study. The uncapacitated facility location problem (UFLP), which has been widely studied in the literature and where the decision variables take 0 or 1 value, is chosen as the test problem. The UFLP is an NP-hard problem that involves decisions on whether to build potential facilities with minimum cost in line with customer demands and customer-facility assignments and is difficult to solve due to its NP-hard problem structure [1,21]. In this context, the main objective of the study is to evaluate the solution performance of the PKO algorithm, which is transformed into a binary structure through transfer functions, on the UFLP.

### Motivation

Proposed in 2024 by Bouaouda et al. [35], the pied kingfisher optimizer (PKO) is an algorithm proposed for solving continuous optimization problems. When the literature is examined, it can be seen that there is no version of the PKO algorithm adapted to binary space. For this reason, in this study, the PKO algorithm is considered and converted to binary using transfer functions. Thus, fourteen different binary PKO versions are proposed in this study. In addition, in order to improve the performance of the algorithm, the Levy flight strategy is added to the algorithm, and the proposed algorithm is called IPKO. The performance of the proposed fourteen different binary IPKO versions is tested on the uncapacitated facility location problem (UFLP). In order to comprehensively evaluate the performance of the proposed fourteen different binary IPKO versions, box plots are presented along with graphs showing the convergence behavior of the algorithms. In addition, Friedman’s mean rank test is applied to determine whether the results obtained are statistically significant. According to the experimental results and statistical analysis, binary IPKO is more successful than the compared algorithms. The results, supported by statistical analysis, show that the proposed binary IPKO is an effective alternative for UFLP with a competitive performance.

The main contributions of this study are summarized below:

- The proposed BinIPKO algorithm has been tested by applying it to the uncapacitated facility location problem (UFLP), which is an optimization problem with a binary solution space.
- The performance of the BinIPKO algorithm was evaluated with different transfer functions, and it was determined that the best results were obtained with the TF1 transfer function. Therefore, only the BinIPKO variant with the TF1 transfer function was used in the comparative analyses.
- The performance of the BinIPKO algorithm was evaluated in comparison with the PSO, GWO, APO, and EEFO algorithms commonly used in the literature.
- The results obtained were analyzed using the Friedman ranking test as well as the TOPSIS and PROMETHEE methods, and it was found that the proposed algorithm demonstrated competitive performance.

## 2. Pied Kingfisher Optimizer (PKO)

The pied kingfisher optimizer (PKO) is a metaheuristic algorithm designed by Bouaouda et al. [35] in 2024 inspired by the perching, hovering, diving, and commensalism behaviors of the pied kingfisher bird. The initial population of the PKO algorithm is initialized as shown in Equation (1).

(1) $$\begin{array}{l} X_{i,j}=\;\;\;\;\;LB+\left(UB-LB\right)\times rand,\\ \;\;i=\;\;\;\;\;1,2,\ldots ,N\;and\;j=1,2,\ldots ,Dim \end{array}$$

where $X_{i,j}$ represents the position of the *i^th^* individual in the *j^th^* dimension, UB is the upper bound, LB is the lower bound, and rand is a random number between 0 and 1. The mathematical modeling of the PKO algorithm consists of the perching and hovering strategy, the diving strategy, and the commensalism phase [35].

### 2.1. Perching and Hovering Strategy

The perching and hovering strategy constitutes the exploration phase of the algorithm. The position of the pied kingfisher is updated according to Equation (2).

(2) $$\begin{array}{c} X_{i}\left(t+1\right)=X_{i}\left(t\right)+\alpha *T\times \left(X_{j}\left(t\right)-X_{i}\left(t\right)\right),\\ i,j=1,2,\ldots ,N\;and\;j\neq i \end{array}$$

Here α is calculated as in Equation (3).

(3) $$\alpha =2\times randn\left(1,Dim\right)-1$$

T represents the perching behavior of the pied kingfisher and is calculated as in Equation (4).

(4) $$T=\left(\exp\left(1\right)-\exp{\left(\frac{t-1}{Max_{Iter}}\right)}^{\frac{1}{BF}}\right)*\cos\left(Crest_{angles}\right)\;Crest_{angles}=2*pi*rand$$

The hovering behavior of the pied kingfisher is calculated as in Equation (5).

(5) $$T=beating_{rate}-\left(\frac{t^{\frac{1}{BF}}}{Max_{Iter^{\frac{1}{BF}}}}\right)\;beating_{rate}=rand*\left(\frac{PKO_{Fitness\left(j\right)}}{PKO_{Fitness\left(i\right)}}\right)$$

where $PKO_{Fitness\left(j\right)}$ shows the fitness value of the *j*th pied kingfisher, while $PKO_{Fitness\left(i\right)}$ shows the fitness value of the *i*th pied kingfisher. *BF* represents the beating factor and has a value of 8. *rand* indicates a random number between 0 and 1.

### 2.2. Diving Strategy

The diving strategy used by the pied kingfisher to catch the fish constitutes the exploitation phase of the algorithm. The mathematical model for this strategy is given in Equation (6).

(6) $$X_{i}\left(t+1\right)=X_{i}\left(t\right)+HA*o*\alpha *\left(b-X_{best}\left(t\right)\right),i=1,2,\ldots ,N\;HA=rand*\left(\frac{PKO_{Fitness\left(i\right)}}{Best_{Fitness}}\right)\;o=exp{\left(\frac{-t}{Max_{Iter}}\right)}^{2}\;b=X_{i}\left(t\right)+o^{2}*randn*X_{best}\left(t\right)$$

### 2.3. Commensalism Phase

The commensalism phase is also defined as the local escape phase of the algorithm. The mathematical model of this phase is given in Equations (7) and (8).

(7) $$X_{i}^{t+1}=\left\{\begin{array}{c} X_{r1}^{t}+o*\alpha *abs\left(X_{i}^{t}-X_{r2}^{t}\right)\;\;\;\;\;\;if\;\;\;\;rand>\left(1-PE\right)\;\;\;\left(a\right)\\ X_{i}^{t}\;\;\;\;\;\;\;\;\;\;\;\;\;\;\;\;\;\;\;\;\;\;\;\;\;\;\;\;\;\;\;\;\;\;\;\;\;\;\;\;\;\;\;\;\;\;\;\;\;\;\;\;\;\;\;\;\;\;\;\;\;\;\;\;otherwise\;\;\;\;\;\;\left(b\right) \end{array}\right.$$

(8) $$\begin{array}{cc} PE= & PE_{max}-\left(PE_{max}-PE_{min}\right)*\left(\frac{t}{Max_{Iter}}\right) \end{array}$$

## 3. Binary Pied Kingfisher Optimizer (BinPKO)

**Figure 1:**
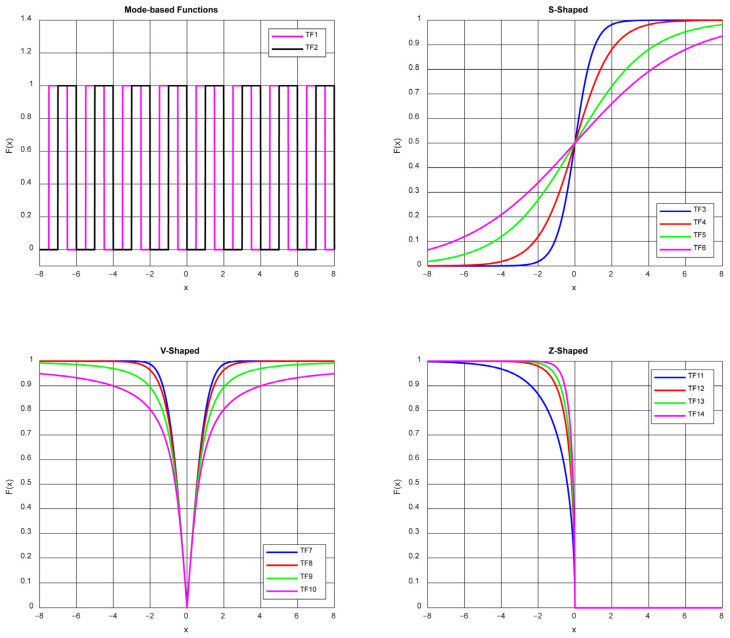
Graphical representations of transfer functions.

The recently proposed PKO algorithm has been designed for solving continuous problems. For this reason, it is not suitable for binary problems and cannot provide solutions. In order for an algorithm to work in binary space, various binary techniques need to be integrated into the algorithm. When such techniques are applied, the output of the algorithm becomes a solution vector consisting only of 0 s and 1 s, which is the desired structure for many binary optimization problems. In the literature, various binary techniques have been developed to adapt continuous algorithms to binary space. In this study, transfer functions that convert the output to binary form while preserving the continuous nature of the algorithm are used [4,36,37]. The transfer functions used in this study are mode functions [17] (TF1 and TF2), S-shaped functions [36] (TF3, TF4, TF5, and TF6), V-shaped functions [36] (TF7, TF8, TF9, and TF10), and Z-shaped functions [32] (TF11, TF12, TF13, and TF14). In order for the PKO algorithm to work in binary space, a total of 14 different transfer functions were used. The equations and visualizations of these functions are given in Table 1 and Figure 1, respectively.

### Levy Flight Strategy

Recently, the Levy flight strategy has been applied to many metaheuristic algorithms to improve their performance. Levy flight alternates between high-frequency short-range exploration and low-frequency long-range exploration to avoid falling into local optimum while searching for optimal solutions over a wide range [38]. Therefore, using Levy flights in many algorithms can increase the diversity of the population distribution and find global optimal solutions faster [38]. Levy flight (LF) is a random walk strategy derived from a non-Gaussian Levy stable distribution based on the power law $L\left(s\right)={\left|s\right|}^{-1-\beta }$ defined on the interval 0<β⩽2 [39]. The mathematical formulation of Levy flight is presented in Equation (9) [39,40].

(9) $$L\left(s,\gamma ,\mu \right)=\left\{\begin{array}{cc} \sqrt{\frac{\gamma }{2\pi }}exp\left[-\frac{\gamma }{2\left(s-\mu \right)}\right]\frac{1}{{\left(s-\mu \right)}^{3/2}} & \;\;\;\;\;\;\;\;if\;\;\;\;0<\mu <s<\infty \\ 0 & if\;\;\;\;s\;\leq 0 \end{array}\right.$$

The computational complexity of the BinIPKO algorithm, considering the population size *N*, dimension *D*, and maximum iteration number *T*, is as shown in Equations (10) and (11).

(10) $$O\left(\mathrm{BinIPKO}\right)=\underset{\mathrm{Initialization}}{\underset{︸}{\left(O\left(N\cdot Dim\right)\right)}}+\underset{\mathrm{Cost}\;\mathrm{function}}{\underset{︸}{\left(O\left(T\cdot N\right)\right)}}+\underset{\mathrm{Updating}\;\mathrm{strategy}}{\underset{︸}{\left(O\left(T\cdot N\cdot Dim\right)\right)}}+\underset{\mathrm{Transfer}\;\mathrm{function}}{\underset{︸}{\left(O\left(T\cdot N\cdot Dim\right)\right)}}+\underset{\mathrm{Levy}\;\mathrm{flight}}{\underset{︸}{\left(O\left(T\cdot N\cdot Dim\right)\right)}}$$

(11) $$\begin{array}{c} O\left(BinIPKO\right)=O\left(NDim+TN+TNDim\right)\\ O\left(BinIPKO\right)≅O\left(TNDim\right) \end{array}$$

Since time-varying transfer functions are executed once in each iteration, the proposed BinIPKO does not cause any change in the computational complexity. Therefore, the computational complexity of BinIPKO is similar to that of the original PKO algorithm. Algorithm 1 presents the pseudocode for the BinIPKO algorithm, which includes all steps created by integrating transfer functions and the LF strategy.
**Algorithm 1:** Binary Improved Pied Kingfisher Optimizer with Levy Flight (BinIPKO)**Input:** Maximum number of iterations *MaxIter*, population size *N*, Beating factor *BF*, Transfer function *TF* **Output:** Binary location of the improved pied kingfisher, and its corresponding fitness value1   Initialize population *X_i_* (*i = 1, 2, …, N*) in continuous space; 2   Binarize the initial population using the transfer function (*TF*); 3   Calculate fitness values of the binary pied kingfisher population; 4   **while** *t < MaxIter + 1* **do** 5   |  **for** i = 1 **to** *N* **do** 6   |  |  **if**
*rand() < 0.8*
**then**;               /* Exploration phase */ 7   |  |  |  **if** *rand() < 0.5* **then** 8   |  |  |  |  Compute *T* using Equation (5): 9   |  |  |  |  *T = BR − (t^1/BF^/MaxIter^1/BF^)* 10 |  |  |  **else** 11 |  |  |  |  Compute *T* using Equation (4): 12 |  |  |  |  *T = (e − e(t−1/MaxIter)^(1/BF)^) · cos(θ)*; 13 |  |  |  Update the position using Equation (2) with Levy Flight: 14 |  |  |  *X′_i_ = X_i_ + α · T · (X_j_ − X_i_) + 0.01 · Levy(1, D)*; 15 |  |  **else**;                      /* Exploitation phase */ 16 |  |  |  Compute *b = X_i_ + o^2^ · N(0,1) · X_best_*; 17 |  |  |  Compute *HA = rand() · f(X_i_)/f(X_best_)*; 18 |  |  |  Update the position using Equation (6) with Levy Flight: 19 |  |  |  *X′_i_ = X_i_ + HA · o · α · (b − X_best_) + 0.01 · Levy(1, D)*; 20 |  |  Apply transfer function *TF* to *X′_i_*; 21 |  |  **if** *f(X′_i_) < f(X_i_)* **then** 22 |  |  |  Replace *X_i_* with *X′_i_*; 23 |  |  **if** *f(X′_i_) < f(X_best_)* **then** 24 |  |  |  Update *X_best_* with *X′_i_*; 25 |  Compute *PE = PE_max_ − (PE_max_ − PE_min_) · t/MaxIter*; 26 |  **for** *i = 1 to N* **do** 27 |  |  **if** *rand() > (1− PE)* **then** 28 |  |  |  Randomly select *r_1_*, *r_2_*; 29 |  |  |  Update position using Equation (7a) with Levy Flight: 30 |  |  |  *X′_i_ = X_r1_ + o · α · |X_i_ − X_r2_| + 0.01 · Levy(1, D)*; 31 |  |  **else** 32 |  |  |  Update position using Equation (7b) with Levy Flight: 33 |  |  |  *X′_i_ = X_i_ + 0.01 · Levy(1, D)*; 34 |  |  Apply transfer function *TF* to *X′_i_*; 35 |  |  Evaluate *f(X′_i_);* 36 |  |  **if** *f(X′_i_) < f(X_i_)* **then** 37 |  |  |  Replace *X_i_* with *X′_i_*; 38 |  |  **if** *f(X′i) < f(X_best_)* **then** 39 |  |  |  Update *X_best_* with *X′_i_*; 40  *t ← t + 1*; 41 **return** *X_best_, f(X_best_)*

## 4. Uncapacitated Facility Location Problem (UFLP)

The main objective of UFLP is to minimize the total cost incurred when deciding on the location of facilities [1]. The total cost includes the costs related to the location of the facilities and the costs related to customer demands [21]. Thus, the number and location of facilities are determined in the most appropriate way to meet customer demands. The mathematical formulation of UFLP is given in Equations (12)–(17) [21,41].

(12) $$\nu \left(P\right)=min\left\{\underset{i\in I}{\sum }\underset{j\in J}{\sum }c_{ij}x_{ij}+\underset{j\in J}{\sum }f_{j}y_{j}\right\}$$

(13) $$\underset{j\in J}{\sum }x_{ij}⩾1,i\in I$$

(14) $$\underset{j\in J}{\sum }y_{j}⩽p$$

(15) $$x_{ij}-y_{j}⩽0,i\in I,j\in J$$

(16) $$x_{ij}\in \left\{0,1\right\},i\in I,j\in J$$

(17) $$y_{j}\in \left\{0,1\right\},j\in J$$

## 5. Experimental Results

In order to evaluate the performance of the BinPKO and BinIPKO versions generated with fourteen different transfer functions, they were applied to 15 different UFLPs in the OR-Lib dataset [42]. The names, sizes, and optimum values of the 15 different problems used in this study are presented in Table 2. 

Cap71, Cap72, Cap73, and Cap74 in Table 2 are classified as small-sized, i.e., 16 × 50, while Cap101, Cap102, Cap103, and Cap104 are classified as medium-sized, i.e., 25 × 50, Cap131, Cap132, Cap133, and Cap134 are classified as large-sized, i.e., 50 × 50, and CapA, CapB, and CapC are classified as very large-sized, i.e., 100 × 1000. For the performance evaluation of the BinPKO and BinIPKO versions created according to the transfer functions on the problems, each BinPKO and BinIPKO version was run 30 times. The population number of the algorithm was set to 40, and the number of iterations was set to 2000. The best, mean, standard deviation (std), and gap values obtained according to these conditions are given in the relevant tables. Also, the formula for the gap value [21,23] is given in Equation (18).

(18) $$Gap=\frac{mean-opt}{opt}\times 100$$

The best, mean, std, and gap results obtained for Cap71, Cap72, Cap73, and Cap74 problems according to fourteen different transfer functions of BinPKO and BinIPKO are given in Table 3.

When Table 3 is examined, the results obtained by both BinPKO and BinIPKO on Cap71, Cap72, Cap73, and Cap74 problems with fourteen different transfer functions successfully reach the best value in all transfer functions except TF11, TF12, TF13, and TF14. The best, mean, std, and gap results obtained for Cap101, Cap102, Cap103, and Cap104 problems with fourteen different transfer functions of BinPKO and BinIPKO are given in Table 4.

When the results in Table 4 are analyzed, results show that the transfer functions TF1, TF2, TF3, TF4, TF7, TF8, TF9, and TF10 successfully reach the best solution for the Cap101 problem for BinPKO. In the Cap102 problem for BinIPKO, the best result is obtained with the transfer functions TF1, TF2, TF3, TF7, TF8, TF9, and TF10. Similarly, in Cap103 and Cap104 problems, TF1, TF2, TF7, TF8, TF8, TF9, and TF10 transfer functions are effective in reaching the best solution. The comparative results of best, mean, std, and gap values of Cap131, Cap132, Cap133, and Cap104 problems according to fourteen different transfer functions of BinPKO and BinIPKO are shown in Table 5.

When the results given in Table 5 are analyzed, it is seen that the best solution for BinPKO is obtained with TF1 and TF2 in Cap131, Cap132, Cap133, and Cap134 problems. In BinIPKO, it is seen that the best solution is obtained with TF1, TF2, and TF8. The comparative results of the best, mean, std and gap values of CapA, CapB, and CapC problems according to fourteen different transfer functions of BinPKO and BinIPKO are presented in Table 6.

**Figure 2:**
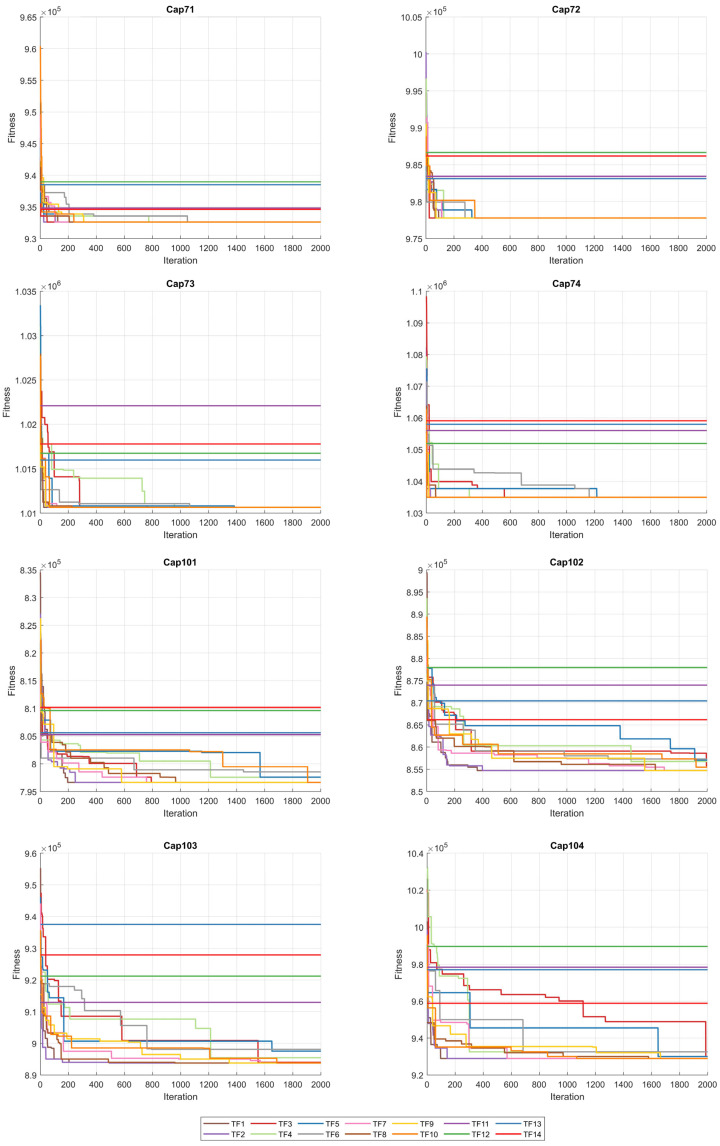
Convergence graphs for Cap71-Cap104 problems according to TFs.

**Figure 3:**
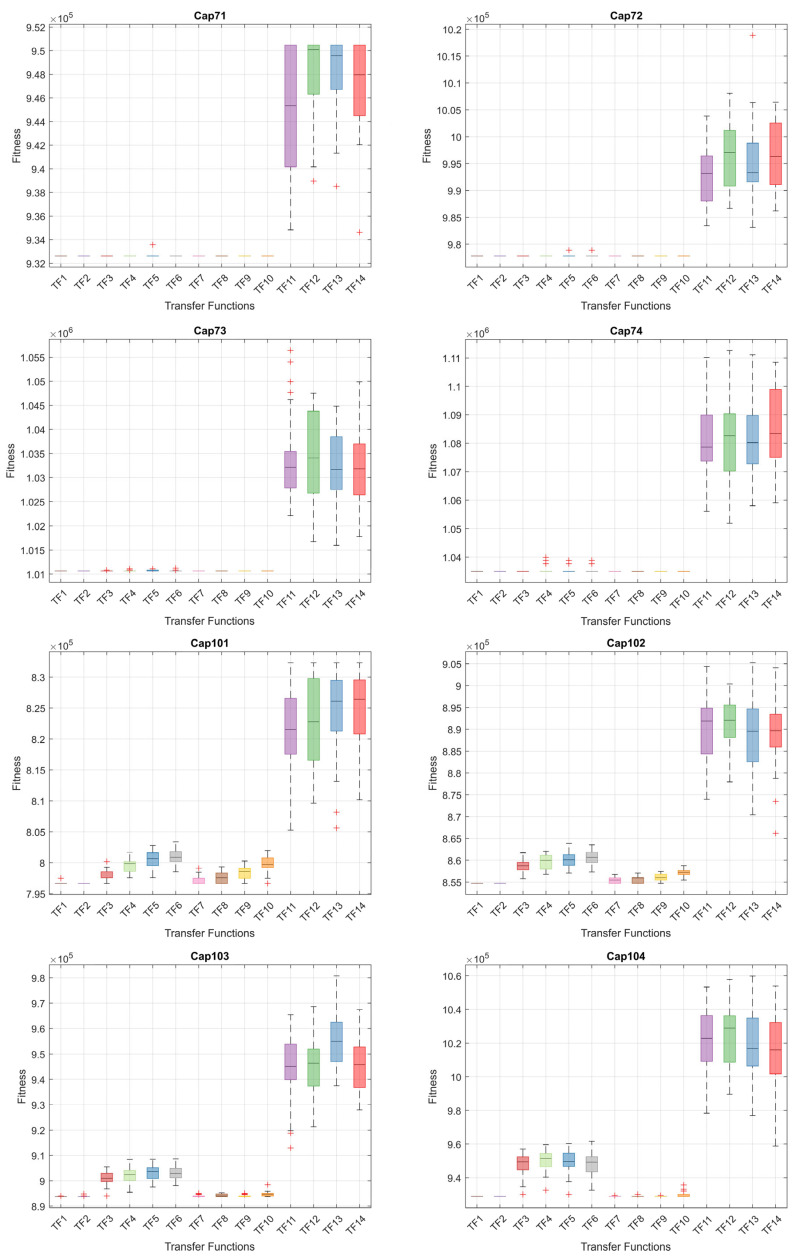
Box plots for Cap71-Cap104 problems by TFs.

**Figure 4:**
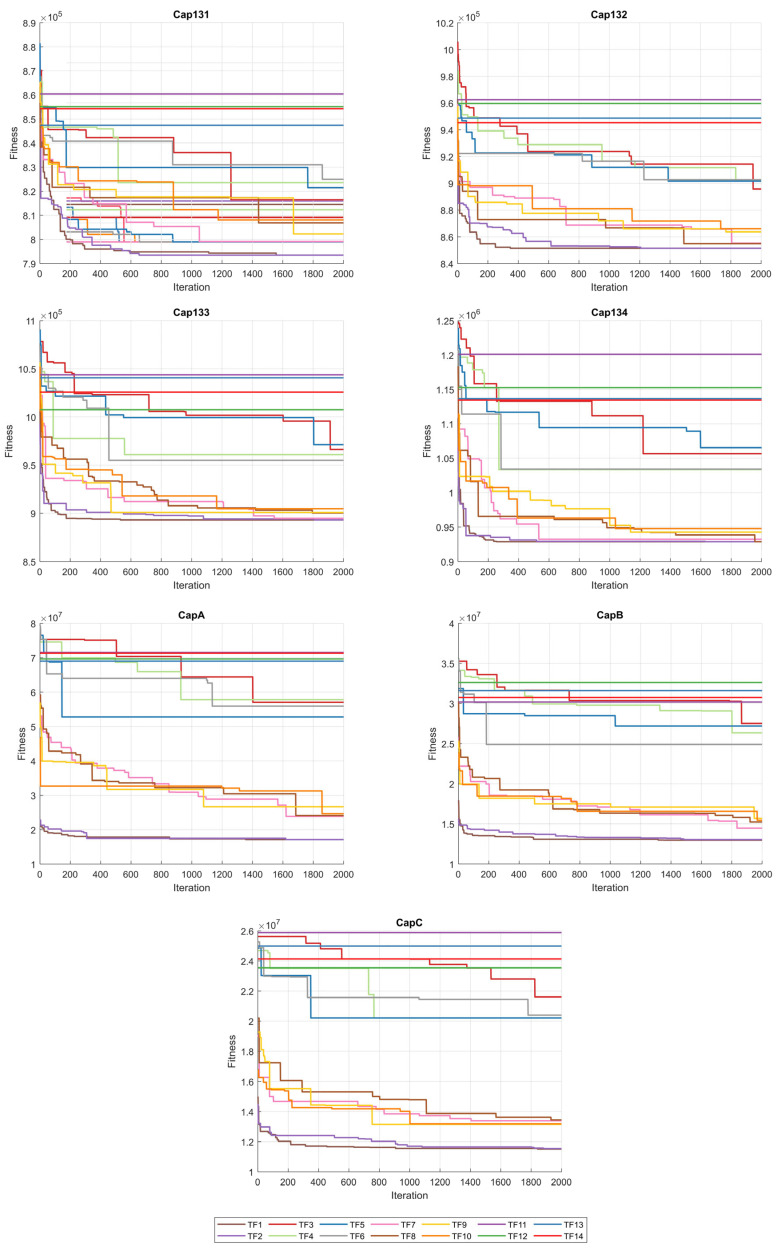
Convergence graphs for Cap131-CapC problems according to TFs.

**Figure 5:**
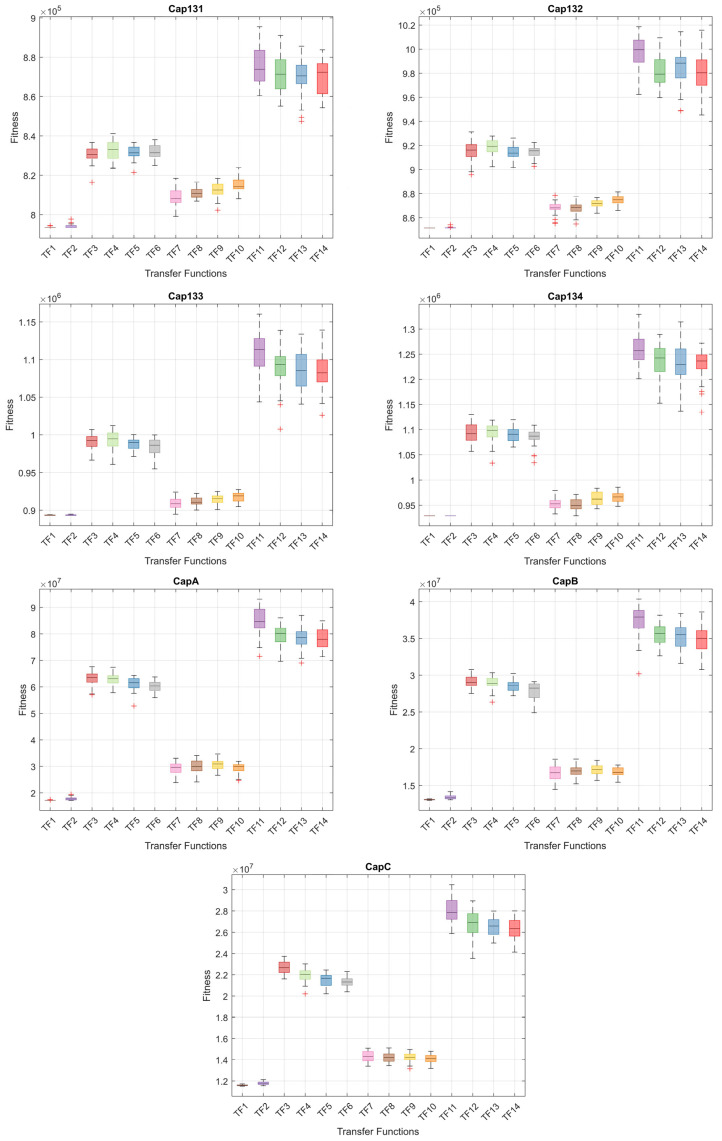
Box plots for Cap131-CapC problems by TFs.

When the results in Table 6 are analyzed, the BinPKO method obtains the best solution with TF1 and TF2 transfer functions in the CapA problem. In the CapB problem, the best solution is obtained with the TF2 transfer function. In the CapC problem, the best solution was obtained with the TF1 transfer function. The BinIPKO method obtained the same value as BinPKO in the CapA problem with the TF1 and TF2 transfer functions, while it obtained a better result than BinPKO in the CapB problem with the TF1 transfer function. In the CapC problem, the BinIPKO method obtained a better result with the TF1 transfer function than the versions obtained with the BinPKO transfer function. According to the transfer functions of BinIPKO, the convergence graphs obtained for Cap71, Cap72, Cap73, Cap74, Cap101, Cap102, Cap103, and Cap104 problems are shown in Figure 2; box plots are shown in Figure 3; convergence graphs obtained for Cap131, Cap132, Cap133, Cap134, CapA, CapB, and CapC problems are shown in Figure 4; and box plots are shown in Figure 5.

The performance rankings of BinIPKO according to the best values obtained from Cap problems with transfer function variants are presented in Table 7, and the performance rankings according to the mean values are presented in Table 8.

In Table 7, the performance of 14 different transfer functions belonging to BinIPKO on various Cap problems are presented comparatively according to their best values. The results in the table show that the transfer functions TF1 achieve the best ranking values in all problems. The TF1 transfer function ranked first with a mean ranking value of 1.00, while the TF2 transfer function ranked second with a mean ranking value of 1.13.

When Table 8 is examined, the ranking achievements of 14 transfer functions of BinIPKO according to their mean values in 15 different Cap problems are compared. The results in the table show that the transfer functions TF1 achieve the lowest ranking in all problems. In particular, TF1 is ranked first since it obtained the best value in all problems, while TF2 is usually ranked second. The TF1 transfer function ranked first with a mean ranking value of 1.07, while the TF2 transfer function ranked second with a mean ranking value of 1.47.

### Comparison of BinIPKO with Literature Algorithms

As a result of the analyses performed on different variants of the BinIPKO algorithm, it was determined that the best performance was obtained with the TF1 transfer function. Therefore, the results presented in this section belong to the TF1 variant and represent the BinIPKO algorithm. In order to analyze the performance of BinIPKO in detail, it is compared with the well-known particle swarm optimization (PSO) [43] and grey wolf optimizer (GWO) [44] algorithms in the literature as well as the recently proposed artificial protozoa optimizer (APO) [45] and electric eel foraging optimization (EEFO) [46] algorithms. The population size is set to 40 and the number of iterations to 2000 for all algorithms. For these algorithms, there are studies in the literature where the transfer functions that provide the best performance are determined. In this context, some studies have determined the most suitable transfer functions; it has been reported that the Z2 transfer function for the PSO algorithm [32], the S4 transfer function for GWO [47], the V2 transfer function for APO [48], and the S1 transfer function for EEFO [49] show the best performance. In addition, the Friedman mean rank statistical test [50], TOPSIS [51], and PROMETHEE [52] methods were used to analyze all results in Table 9. The results obtained based on the best-performing transfer functions according to the algorithms and the results of BinIPKO are presented in Table 9. Furthermore, the performance ranking of BinIPKO and some algorithms in the literature according to the mean values obtained from Cap problems is presented in Table 10.

In Table 9, the BinIPKO results obtained from Cap problems are compared with the results of the BinPKO, EEFO, PSO, APO, and GWO algorithms. As a result of the comparison, the BinIPKO algorithm achieved the highest success rate and ranked first. The BinPKO algorithm followed, ranked second. The GWO algorithm ranked third, while the APO algorithm ranked fourth. The EEFO algorithm ranked fifth, and the PSO algorithm, which showed the lowest performance, ranked last.

Table 10 shows the ranking performance of the algorithms according to their mean values based on the results obtained from UFLPs. The BinIPKO algorithm ranked first and was found to be a successful method. The BinPKO algorithm ranked second, while the GWO algorithm ranked third, the APO algorithm ranked fourth, and the EEFO algorithm ranked fifth. The PSO algorithm showed the lowest performance. 

The contributions of the study can be summarized as follows based on the findings:

- BinIPKO has been found to achieve the best results in Cap problems using the TF1 transfer function.
- The TF1 variant of BinIPKO ranked first in Cap problems and demonstrated more successful performance than the algorithms compared.
- The analyses were supported not only by the best solution values but also by statistical significance tests provided by the Friedman test, as well as multi-criteria evaluations performed using the TOPSIS and PROMETHEE methods.

## 6. Discussion

In this study, the performance of the proposed BinIPKO algorithm for the UFLP is analyzed in detail. The BinIPKO algorithm is first tested on Cap problems with fourteen different transfer functions. According to both the best and the mean values obtained, BinIPKO obtained the best results with the TF1 transfer function. For this reason, the literature comparisons were carried out on the TF1 variant of BinIPKO. As a result of the experimental analysis, the BinIPKO algorithm ranked first in the Cap problems and achieved better success than the compared methods. The findings are supported not only by the best solution values but also by statistical analysis and multi-criteria decision-making methods. The Friedman mean rank test showed that BinIPKO was more successful by producing statistically significant results in terms of mean rank. In addition, TOPSIS and PROMETHEE methods were applied to evaluate the overall success of the algorithms. The results obtained with both methods show that BinIPKO is more successful than the compared algorithms. As a result of all the experimental analyses, it is observed that the BinIPKO algorithm is a more competitive method for the UFLP than the compared algorithms.

## 7. Conclusions

In this study, the performance of the PKO algorithm is evaluated by adapting it to the binary solution space for UFLP. For this purpose, fourteen different transfer functions were integrated into the PKO algorithm, and fourteen different binary variants of the algorithm were generated. The general name of these variants is called BinPKO. In addition, a Levy flight strategy was added to the algorithm to improve the performance of BinPKO. The overall name of the fourteen different variants created in this way is called BinIPKO. Each BinPKO and BinIPKO variant was applied to fifteen different Cap problems, and their performance was analyzed. When the performance of both algorithms is analyzed according to the mean value, it is observed that although they achieve the same results in Cap71, Cap72, Cap73, Cap74, and Cap104 problems, BinIPKO is more successful in the remaining problems. In particular, TF1 ranked first in almost all problems, while TF2 generally ranked second. Although the other transfer functions were able to provide competitive results on some problems, they lagged behind TF1 and TF2 in the overall rankings. The transfer functions TF11, TF12, TF13, and TF14 were ranked last in both best and mean rankings. In addition, when the performance of BinIPKO is compared with the well-known PSO and GWO algorithms in the literature as well as the recently proposed APO and EEFO algorithms, it is seen that BinIPKO is more successful by ranking first according to the mean ranking statistic. All the results obtained from the study show that the choice of transfer function has a decisive effect on algorithm performance in binary optimization problems. In this respect, the TF1 transfer function stands out by performing well in terms of both solution quality and stability when used with the IPKO algorithm.

## Figures and Tables

**Table 1:** Transfer functions and mathematical formulas used in the study.

TF Name	Transfer Functions
TF1	$T\left(x\right)=\mathrm{mod}\left(\mathrm{round}(\mathrm{mod}\left(x,2\right),\;2\right);$
TF2	$T\left(x\right)=\mathrm{mod}\left(\mathrm{floor}\left(x\right),\;2\right);$
TF3	$T\left(x\right)=\frac{1}{1+e^{-2x}}$
TF4	$T\left(x\right)=\frac{1}{1+e^{-x}}\;$
TF5	$T\left(x\right)=\frac{1}{1+e^{-\frac{x}{2}}}$
TF6	$T\left(x\right)=\frac{1}{1+e^{-\frac{x}{3}}}$
TF7	$T\left(x\right)=\left|erf\left(\frac{\sqrt{\pi }}{2}\right)x\right|$
TF8	$T\left(x\right)=\left|tanh\left(x\right)\right|$
TF9	$T\left(x\right)=\left|\frac{\left(x\right)}{\sqrt{1+x^{2}}}\right|$
TF10	$T\left(x\right)=\left|\frac{2}{\pi }arctan\left(\frac{\pi }{2}\right)x\right|$
TF11	$T\left(x\right)=\sqrt{1-2^{x}}$
TF12	$T\left(x\right)=\sqrt{1-5^{x}}$
TF13	$T\left(x\right)=\sqrt{1-8^{x}}$
TF14	$T\left(x\right)=\sqrt{1-20^{x}}$

**Table 2:** Data information about the problem.

Problems	Dimension	Fitness
Cap71	16 × 50	9.32616 × 10^5^
Cap72	16 × 50	9.77799 × 10^5^
Cap73	16 × 50	1.01064 × 10^6^
Cap74	16 × 50	1.03498 × 10^6^
Cap101	25 × 50	7.96648 × 10^5^
Cap102	25 × 50	8.54704 × 10^5^
Cap103	25 × 50	8.93782 × 10^5^
Cap104	25 × 50	9.28942 × 10^5^
Cap131	50 × 50	7.93440 × 10^5^
Cap132	50 × 50	8.51495 × 10^5^
Cap133	50 × 50	8.93077 × 10^5^
Cap134	50 × 50	9.28942 × 10^5^
CapA	100 × 1000	1.71565 × 10^7^
CapB	100 × 1000	1.29791 × 10^7^
CapC	100 × 1000	1.15056 × 10^7^

**Table 3:** Results obtained for Cap71, Cap72, Cap73, and Cap74 problems according to TFs.

TF	Criteria	Cap71	Cap72	Cap73	Cap74
BinPKO-TF1	Best	9.32616 × 10^5^	9.77799 × 10^5^	1.01064 × 10^6^	1.03498 × 10^6^
	Mean	9.32616 × 10^5^	9.77799 × 10^5^	1.01064 × 10^6^	1.03498 × 10^6^
	Std	0.00000 × 10^0^	4.73622 × 10^−10^	4.73622 × 10^−10^	3.55216 × 10^−10^
	Gap	0.00000 × 10^0^	0.00000 × 10^0^	0.00000 × 10^0^	0.00000 × 10^0^
BinIPKO-TF1	Best	9.32616 × 10^5^	9.77799 × 10^5^	1.01064 × 10^6^	1.03498 × 10^6^
	Mean	9.32616 × 10^5^	9.77799 × 10^5^	1.01064 × 10^6^	1.03498 × 10^6^
	Std	0.00000 × 10^0^	4.73622 × 10^−10^	4.73622 × 10^−10^	3.55216 × 10^−10^
	Gap	0.00000 × 10^0^	0.00000 × 10^0^	0.00000 × 10^0^	0.00000 × 10^0^
BinPKO-TF2	Best	9.32616 × 10^5^	9.77799 × 10^5^	1.01064 × 10^6^	1.03498 × 10^6^
	Mean	9.32616 × 10^5^	9.77799 × 10^5^	1.01064 × 10^6^	1.03498 × 10^6^
	Std	0.00000 × 10^0^	4.73622 × 10^−10^	4.73622 × 10^−10^	3.55216 × 10^−10^
	Gap	0.00000 × 10^0^	0.00000 × 10^0^	0.00000 × 10^0^	0.00000 × 10^0^
BinIPKO-TF2	Best	9.32616 × 10^5^	9.77799 × 10^5^	1.01064 × 10^6^	1.03498 × 10^6^
	Mean	9.32616 × 10^5^	9.77799 × 10^5^	1.01064 × 10^6^	1.03498 × 10^6^
	Std	0.00000 × 10^0^	4.73622 × 10^−10^	4.73622 × 10^−10^	3.55216 × 10^−10^
	Gap	0.00000 × 10^0^	0.00000 × 10^0^	0.00000 × 10^0^	0.00000 × 10^0^
BinPKO-TF3	Best	9.32616 × 10^5^	9.77799 × 10^5^	1.01064 × 10^6^	1.03498 × 10^6^
	Mean	9.32616 × 10^5^	9.77799 × 10^5^	1.01066 × 10^6^	1.03525 × 10^6^
	Std	0.00000 × 10^0^	4.73622 × 10^−10^	5.08687 × 10^1^	8.36083 × 10^2^
	Gap	0.00000 × 10^0^	0.00000 × 10^0^	1.64957 × 10^−3^	2.64750 × 10^−2^
BinIPKO-TF3	Best	9.32616 × 10^5^	9.77799 × 10^5^	1.01064 × 10^6^	1.03498 × 10^6^
	Mean	9.32616 × 10^5^	9.77799 × 10^5^	1.01065 × 10^6^	1.03498 × 10^6^
	Std	0.00000 × 10^0^	4.73622 × 10^−10^	4.22963 × 10^1^	3.55216 × 10^−10^
	Gap	0.00000 × 10^0^	0.00000 × 10^0^	1.09971 × 10^−3^	0.00000 × 10^0^
BinPKO-TF4	Best	9.32616 × 10^5^	9.77799 × 10^5^	1.01064 × 10^6^	1.03498 × 10^6^
	Mean	9.32616 × 10^5^	9.77799 × 10^5^	1.01067 × 10^6^	1.03543 × 10^6^
	Std	0.00000 × 10^0^	4.73622 × 10^−10^	6.78250 × 10^1^	1.03863 × 10^3^
	Gap	0.00000 × 10^0^	0.00000 × 10^0^	3.29914 × 10^−3^	4.41250 × 10^−2^
BinIPKO-TF4	Best	9.32616 × 10^5^	9.77799 × 10^5^	1.01064 × 10^6^	1.03498 × 10^6^
	Mean	9.32616 × 10^5^	9.77799 × 10^5^	1.01069 × 10^6^	1.03564 × 10^6^
	Std	0.00000 × 10^0^	4.73622 × 10^−10^	9.83619 × 10^1^	1.39491 × 10^3^
	Gap	0.00000 × 10^0^	0.00000 × 10^0^	4.70485 × 10^−3^	6.36858 × 10^−2^
BinPKO-TF5	Best	9.32616 × 10^5^	9.77799 × 10^5^	1.01064 × 10^6^	1.03498 × 10^6^
	Mean	9.32648 × 10^5^	9.77835 × 10^5^	1.01070 × 10^6^	1.03587 × 10^6^
	Std	1.74021 × 10^2^	1.96614 × 10^2^	1.34274 × 10^2^	1.44429 × 10^3^
	Gap	3.40673 × 10^−3^	3.67117 × 10^−3^	5.86669 × 10^−3^	8.65398 × 10^−2^
BinIPKO-TF5	Best	9.32616 × 10^5^	9.77799 × 10^5^	1.01064 × 10^6^	1.03498 × 10^6^
	Mean	9.32648 × 10^5^	9.77871 × 10^5^	1.01069 × 10^6^	1.03569 × 10^6^
	Std	1.74021 × 10^2^	2.73218 × 10^2^	1.00274 × 10^2^	1.34036 × 10^3^
	Gap	3.40673 × 10^−3^	7.34234 × 10^−3^	5.25471 × 10^−3^	6.90170 × 10^−2^
BinPKO-TF6	Best	9.32616 × 10^5^	9.77799 × 10^5^	1.01064 × 10^6^	1.03498 × 10^6^
	Mean	9.32648 × 10^5^	9.77835 × 10^5^	1.01067 × 10^6^	1.03538 × 10^6^
	Std	1.74021 × 10^2^	1.96614 × 10^2^	6.31922 × 10^1^	1.06009 × 10^3^
	Gap	3.40673 × 10^−3^	3.67117 × 10^−3^	2.74929 × 10^−3^	3.89210 × 10^−2^
BinIPKO-TF6	Best	9.32616 × 10^5^	9.77799 × 10^5^	1.01064 × 10^6^	1.03498 × 10^6^
	Mean	9.32616 × 10^5^	9.77871 × 10^5^	1.01069 × 10^6^	1.03556 × 10^6^
	Std	0.00000 × 10^0^	2.73218 × 10^2^	1.22291 × 10^2^	1.20616 × 10^3^
	Gap	0.00000 × 10^0^	7.34234 × 10^−3^	5.25471 × 10^−3^	5.65710 × 10^−2^
BinPKO-TF7	Best	9.32616 × 10^5^	9.77799 × 10^5^	1.01064 × 10^6^	1.03498 × 10^6^
	Mean	9.32616 × 10^5^	9.77799 × 10^5^	1.01064 × 10^6^	1.03498 × 10^6^
	Std	0.00000 × 10^0^	4.73622 × 10^−10^	4.73622 × 10^−10^	3.55216 × 10^−10^
	Gap	0.00000 × 10^0^	0.00000 × 10^0^	0.00000 × 10^0^	0.00000 × 10^0^
BinIPKO-TF7	Best	9.32616 × 10^5^	9.77799 × 10^5^	1.01064 × 10^6^	1.03498 × 10^6^
	Mean	9.32616 × 10^5^	9.77799 × 10^5^	1.01064 × 10^6^	1.03498 × 10^6^
	Std	0.00000 × 10^0^	4.73622 × 10^−10^	4.73622 × 10^−10^	3.55216 × 10^−10^
	Gap	0.00000 × 10^0^	0.00000 × 10^0^	0.00000 × 10^0^	0.00000 × 10^0^
BinPKO-TF8	Best	9.32616 × 10^5^	9.77799 × 10^5^	1.01064 × 10^6^	1.03498 × 10^6^
	Mean	9.32616 × 10^5^	9.77799 × 10^5^	1.01064 × 10^6^	1.03498 × 10^6^
	Std	0.00000 × 10^0^	4.73622 × 10^−10^	4.73622 × 10^−10^	3.55216 × 10^−10^
	Gap	0.00000 × 10^0^	0.00000 × 10^0^	0.00000 × 10^0^	0.00000 × 10^0^
BinIPKO-TF8	Best	9.32616 × 10^5^	9.77799 × 10^5^	1.01064 × 10^6^	1.03498 × 10^6^
	Mean	9.32616 × 10^5^	9.77799 × 10^5^	1.01064 × 10^6^	1.03498 × 10^6^
	Std	0.00000 × 10^0^	4.73622 × 10^−10^	4.73622 × 10^−10^	3.55216 × 10^−10^
	Gap	0.00000 × 10^0^	0.00000 × 10^0^	0.00000 × 10^0^	0.00000 × 10^0^
BinPKO-TF9	Best	9.32616 × 10^5^	9.77799 × 10^5^	1.01064 × 10^6^	1.03498 × 10^6^
	Mean	9.32616 × 10^5^	9.77799 × 10^5^	1.01064 × 10^6^	1.03498 × 10^6^
	Std	0.00000 × 10^0^	4.73622 × 10^−10^	4.73622 × 10^−10^	3.55216 × 10^−10^
	Gap	0.00000 × 10^0^	0.00000 × 10^0^	0.00000 × 10^0^	0.00000 × 10^0^
BinIPKO-TF9	Best	9.32616 × 10^5^	9.77799 × 10^5^	1.01064 × 10^6^	1.03498 × 10^6^
	Mean	9.32616 × 10^5^	9.77799 × 10^5^	1.01064 × 10^6^	1.03498 × 10^6^
	Std	0.00000 × 10^0^	4.73622 × 10^−10^	4.73622 × 10^−10^	3.55216 × 10^−10^
	Gap	0.00000 × 10^0^	0.00000 × 10^0^	0.00000 × 10^0^	0.00000 × 10^0^
BinPKO-TF10	Best	9.32616 × 10^5^	9.77799 × 10^5^	1.01064 × 10^6^	1.03498 × 10^6^
	Mean	9.32616 × 10^5^	9.77799 × 10^5^	1.01064 × 10^6^	1.03498 × 10^6^
	Std	0.00000 × 10^0^	4.73622 × 10^−10^	4.73622 × 10^−10^	3.55216 × 10^−10^
	Gap	0.00000 × 10^0^	0.00000 × 10^0^	0.00000 × 10^0^	0.00000 × 10^0^
BinIPKO-TF10	Best	9.32616 × 10^5^	9.77799 × 10^5^	1.01064 × 10^6^	1.03498 × 10^6^
	Mean	9.32616 × 10^5^	9.77799 × 10^5^	1.01064 × 10^6^	1.03498 × 10^6^
	Std	0.00000 × 10^0^	4.73622 × 10^−10^	4.73622 × 10^−10^	3.55216 × 10^−10^
	Gap	0.00000 × 10^0^	0.00000 × 10^0^	0.00000 × 10^0^	0.00000 × 10^0^
BinPKO-TF11	Best	9.38355 × 10^5^	9.86019 × 10^5^	1.01477 × 10^6^	1.05663 × 10^6^
	Mean	9.47109 × 10^5^	9.94422 × 10^5^	1.03341 × 10^6^	1.08369 × 10^6^
	Std	3.79165 × 10^3^	5.15366 × 10^3^	7.34087 × 10^3^	1.40876 × 10^4^
	Gap	1.55406 × 10^0^	1.70001 × 10^0^	2.25323 × 10^0^	4.70683 × 10^0^
BinIPKO-TF11	Best	9.34829 × 10^5^	9.83429 × 10^5^	1.02209 × 10^6^	1.05601 × 10^6^
	Mean	9.45048 × 10^5^	9.92778 × 10^5^	1.03367 × 10^6^	1.08113 × 10^6^
	Std	5.41823 × 10^3^	5.70021 × 10^3^	9.40720 × 10^3^	1.41690 × 10^4^
	Gap	1.33304 × 10^0^	1.53191 × 10^0^	2.27893 × 10^0^	4.45981 × 10^0^
BinPKO-TF12	Best	9.35445 × 10^5^	9.82726 × 10^5^	1.01551 × 10^6^	1.05622 × 10^6^
	Mean	9.47653 × 10^5^	9.95695 × 10^5^	1.03169 × 10^6^	1.07647 × 10^6^
	Std	3.94209 × 10^3^	6.29942 × 10^3^	8.71690 × 10^3^	1.20317 × 10^4^
	Gap	1.61240 × 10^0^	1.83018 × 10^0^	2.08317 × 10^0^	4.00862 × 10^0^
BinIPKO-TF12	Best	9.38953 × 10^5^	9.86665 × 10^5^	1.01672 × 10^6^	1.05192 × 10^6^
	Mean	9.48024 × 10^5^	9.97107 × 10^5^	1.03437 × 10^6^	1.08178 × 10^6^
	Std	3.58929 × 10^3^	6.83465 × 10^3^	8.94900 × 10^3^	1.46645 × 10^4^
	Gap	1.65215 × 10^0^	1.97461 × 10^0^	2.34786 × 10^0^	4.52243 × 10^0^
BinPKO-TF13	Best	9.40562 × 10^5^	9.82373 × 10^5^	1.01485 × 10^6^	1.06628 × 10^6^
	Mean	9.47843 × 10^5^	9.95201 × 10^5^	1.03034 × 10^6^	1.08733 × 10^6^
	Std	3.41213 × 10^3^	6.63002 × 10^3^	8.76105 × 10^3^	1.23636 × 10^4^
	Gap	1.63275 × 10^0^	1.77969 × 10^0^	1.94952 × 10^0^	5.05886 × 10^0^
BinIPKO-TF13	Best	9.38515 × 10^5^	9.83122 × 10^5^	1.01596 × 10^6^	1.05796 × 10^6^
	Mean	9.47743 × 10^5^	9.94983 × 10^5^	1.03209 × 10^6^	1.08144 × 10^6^
	Std	3.53634 × 10^3^	7.49982 × 10^3^	7.57757 × 10^3^	1.30608 × 10^4^
	Gap	1.62207 × 10^0^	1.75740 × 10^0^	2.12185 × 10^0^	4.48924 × 10^0^
BinPKO-TF14	Best	9.39182 × 10^5^	9.87137 × 10^5^	1.01081 × 10^6^	1.05346 × 10^6^
	Mean	9.49295 × 10^5^	9.96150 × 10^5^	1.02891 × 10^6^	1.08107 × 10^6^
	Std	2.42321 × 10^3^	5.38402 × 10^3^	8.80615 × 10^3^	1.46911 × 10^4^
	Gap	1.78840 × 10^0^	1.87668 × 10^0^	1.80766 × 10^0^	4.45343 × 10^0^
BinIPKO-TF14	Best	9.34623 × 10^5^	9.86182 × 10^5^	1.01778 × 10^6^	1.05906 × 10^6^
	Mean	9.47136 × 10^5^	9.96615 × 10^5^	1.03209 × 10^6^	1.08492 × 10^6^
	Std	3.90886 × 10^3^	6.30017 × 10^3^	7.96827 × 10^3^	1.45749 × 10^4^
	Gap	1.55691 × 10^0^	1.92427 × 10^0^	2.12180 × 10^0^	4.82572 × 10^0^

**Table 4:** Results obtained for Cap101, Cap102, Cap103, and Cap104 problems according to TFs.

TF	Criteria	Cap101	Cap102	Cap103	Cap104
BinPKO-TF1	Best	7.96648 × 10^5^	8.54704 × 10^5^	8.93782 × 10^5^	9.28942 × 10^5^
	Mean	7.96763 × 10^5^	8.54782 × 10^5^	8.94035 × 10^5^	9.28942 × 10^5^
	Std	2.97441 × 10^2^	2.98455 × 10^2^	4.42600 × 10^2^	0.00000 × 10^0^
	Gap	1.43984 × 10^−2^	9.14332 × 10^−3^	2.82751 × 10^−2^	0.00000 × 10^0^
BinIPKO-TF1	Best	7.96648 × 10^5^	8.54704 × 10^5^	8.93782 × 10^5^	9.28942 × 10^5^
	Mean	7.96677 × 10^5^	8.54704 × 10^5^	8.93790 × 10^5^	9.28942 × 10^5^
	Std	1.57066 × 10^2^	5.92027 × 10^−10^	4.12663 × 10^1^	0.00000 × 10^0^
	Gap	3.59961 × 10^−3^	0.00000 × 10^0^	8.42953 × 10^−4^	0.00000 × 10^0^
BinPKO-TF2	Best	7.96648 × 10^5^	8.54704 × 10^5^	8.93782 × 10^5^	9.28942 × 10^5^
	Mean	7.97134 × 10^5^	8.55607 × 10^5^	8.94394 × 10^5^	9.28942 × 10^5^
	Std	5.69246 × 10^2^	1.46409 × 10^3^	1.09232 × 10^3^	0.00000 × 10^0^
	Gap	6.09272 × 10^−2^	1.05576 × 10^−1^	6.84515 × 10^−2^	0.00000 × 10^0^
BinIPKO-TF2	Best	7.96648 × 10^5^	8.54704 × 10^5^	8.93782 × 10^5^	9.28942 × 10^5^
	Mean	7.96648 × 10^5^	8.54704 × 10^5^	8.93839 × 10^5^	9.28942 × 10^5^
	Std	0.00000 × 10^0^	5.92027 × 10^−10^	1.94379 × 10^2^	0.00000 × 10^0^
	Gap	0.00000 × 10^0^	0.00000 × 10^0^	6.32938 × 10^−3^	0.00000 × 10^0^
BinPKO-TF3	Best	7.96648 × 10^5^	8.54704 × 10^5^	8.94574 × 10^5^	9.36052 × 10^5^
	Mean	7.97815 × 10^5^	8.57757 × 10^5^	9.00544 × 10^5^	9.48694 × 10^5^
	Std	5.30133 × 10^2^	1.32310 × 10^3^	2.78733 × 10^3^	6.06607 × 10^3^
	Gap	1.46472 × 10^−1^	3.57214 × 10^−1^	7.56598 × 10^−1^	2.12635 × 10^0^
BinIPKO-TF3	Best	7.96648 × 10^5^	8.55781 × 10^5^	8.94008 × 10^5^	9.30027 × 10^5^
	Mean	7.97924 × 10^5^	8.58685 × 10^5^	9.00853 × 10^5^	9.47766 × 10^5^
	Std	8.14642 × 10^2^	1.35516 × 10^3^	2.54668 × 10^3^	6.44339 × 10^3^
	Gap	1.60162 × 10^−1^	4.65765 × 10^−1^	7.91095 × 10^−1^	2.02646 × 10^0^
BinPKO-TF4	Best	7.96648 × 10^5^	8.56113 × 10^5^	8.98406 × 10^5^	9.30027 × 10^5^
	Mean	7.99657 × 10^5^	8.59438 × 10^5^	9.02626 × 10^5^	9.50742 × 10^5^
	Std	9.89740 × 10^2^	1.67411 × 10^3^	2.79217 × 10^3^	6.38111 × 10^3^
	Gap	3.77615 × 10^−1^	5.53822 × 10^−1^	9.89539 × 10^−1^	2.34676 × 10^0^
BinIPKO-TF4	Best	7.97582 × 10^5^	8.56767 × 10^5^	8.95462 × 10^5^	9.32527 × 10^5^
	Mean	7.99593 × 10^5^	8.59594 × 10^5^	9.02068 × 10^5^	9.50128 × 10^5^
	Std	1.05738 × 10^3^	1.60487 × 10^3^	3.03706 × 10^3^	6.26330 × 10^3^
	Gap	3.69656 × 10^−1^	5.72068 × 10^−1^	9.27056 × 10^−1^	2.28068 × 10^0^
BinPKO-TF5	Best	7.97657 × 10^5^	8.56719 × 10^5^	8.94008 × 10^5^	9.35592 × 10^5^
	Mean	8.00448 × 10^5^	8.60400 × 10^5^	9.01864 × 10^5^	9.48338 × 10^5^
	Std	1.32470 × 10^3^	2.08137 × 10^3^	3.84782 × 10^3^	5.34060 × 10^3^
	Gap	4.76890 × 10^−1^	6.66406 × 10^−1^	9.04249 × 10^−1^	2.08800 × 10^0^
BinIPKO-TF5	Best	7.97602 × 10^5^	8.57049 × 10^5^	8.97532 × 10^5^	9.30027 × 10^5^
	Mean	8.00635 × 10^5^	8.59925 × 10^5^	9.03252 × 10^5^	9.49339 × 10^5^
	Std	1.29206 × 10^3^	1.86989 × 10^3^	2.65849 × 10^3^	6.39045 × 10^3^
	Gap	5.00366 × 10^−1^	6.10793 × 10^−1^	1.05957 × 10^0^	2.19572 × 10^0^
BinPKO-TF6	Best	7.97582 × 10^5^	8.56004 × 10^5^	8.98447 × 10^5^	9.35106 × 10^5^
	Mean	8.00548 × 10^5^	8.60387 × 10^5^	9.02766 × 10^5^	9.49515 × 10^5^
	Std	1.29314 × 10^3^	1.99052 × 10^3^	2.49645 × 10^3^	5.33981 × 10^3^
	Gap	4.89517 × 10^−1^	6.64924 × 10^−1^	1.00516 × 10^0^	2.21471 × 10^0^
BinIPKO-TF6	Best	7.98535 × 10^5^	8.57308 × 10^5^	8.98103 × 10^5^	9.32527 × 10^5^
	Mean	8.00971 × 10^5^	8.60496 × 10^5^	9.02884 × 10^5^	9.48383 × 10^5^
	Std	1.10792 × 10^3^	1.69721 × 10^3^	2.64880 × 10^3^	5.90283 × 10^3^
	Gap	5.42537 × 10^−1^	6.77693 × 10^−1^	1.01838 × 10^0^	2.09279 × 10^0^
BinPKO-TF7	Best	7.96648 × 10^5^	8.54704 × 10^5^	8.93782 × 10^5^	9.28942 × 10^5^
	Mean	7.97090 × 10^5^	8.55800 × 10^5^	8.94324 × 10^5^	9.29204 × 10^5^
	Std	5.43334 × 10^2^	8.94071 × 10^2^	6.06135 × 10^2^	6.34413 × 10^2^
	Gap	5.53771 × 10^−2^	1.28183 × 10^−1^	6.06139 × 10^−2^	2.82698 × 10^−2^
BinIPKO-TF7	Best	7.96648 × 10^5^	8.54704 × 10^5^	8.93782 × 10^5^	9.28942 × 10^5^
	Mean	7.97234 × 10^5^	8.55352 × 10^5^	8.93978 × 10^5^	9.28959 × 10^5^
	Std	6.56207 × 10^2^	7.08176 × 10^2^	3.29505 × 10^2^	9.47971 × 10^1^
	Gap	7.34806 × 10^−2^	7.57417 × 10^−2^	2.19351 × 10^−2^	1.86314 × 10^−3^
BinPKO-TF8	Best	7.96648 × 10^5^	8.54704 × 10^5^	8.93782 × 10^5^	9.28942 × 10^5^
	Mean	7.97347 × 10^5^	8.55745 × 10^5^	8.94197 × 10^5^	9.29250 × 10^5^
	Std	6.57000 × 10^2^	7.53952 × 10^2^	5.12541 × 10^2^	9.21515 × 10^2^
	Gap	8.77254 × 10^−2^	1.21774 × 10^−1^	4.64226 × 10^−2^	3.31310 × 10^−2^
BinIPKO-TF8	Best	7.96648 × 10^5^	8.54704 × 10^5^	8.93782 × 10^5^	9.28942 × 10^5^
	Mean	7.97584 × 10^5^	8.55682 × 10^5^	8.94254 × 10^5^	9.28978 × 10^5^
	Std	7.97502 × 10^2^	6.87482 × 10^2^	4.82851 × 10^2^	1.98056 × 10^2^
	Gap	1.17438 × 10^−1^	1.14407 × 10^−1^	5.28358 × 10^−2^	3.89260 × 10^−3^
BinPKO-TF9	Best	7.96648 × 10^5^	8.54704 × 10^5^	8.93782 × 10^5^	9.28942 × 10^5^
	Mean	7.98122 × 10^5^	8.56122 × 10^5^	8.94688 × 10^5^	9.29322 × 10^5^
	Std	9.88121 × 10^2^	8.92035 × 10^2^	9.76051 × 10^2^	1.21407 × 10^3^
	Gap	1.84923 × 10^−1^	1.65842 × 10^−1^	1.01407 × 10^−1^	4.09077 × 10^−2^
BinIPKO-TF9	Best	7.96648 × 10^5^	8.54704 × 10^5^	8.93782 × 10^5^	9.28942 × 10^5^
	Mean	7.98369 × 10^5^	8.56124 × 10^5^	8.94118 × 10^5^	9.28959 × 10^5^
	Std	1.04651 × 10^3^	8.38228 × 10^2^	3.72978 × 10^2^	9.47971 × 10^1^
	Gap	2.15993 × 10^−1^	1.66137 × 10^−1^	3.75407 × 10^−2^	1.86314 × 10^−3^
BinPKO-TF10	Best	7.96648 × 10^5^	8.54704 × 10^5^	8.93782 × 10^5^	9.28942 × 10^5^
	Mean	7.99617 × 10^5^	8.57219 × 10^5^	8.94998 × 10^5^	9.29911 × 10^5^
	Std	1.60573 × 10^3^	1.04605 × 10^3^	1.01691 × 10^3^	1.68371 × 10^3^
	Gap	3.72679 × 10^−1^	2.94181 × 10^−1^	1.36053 × 10^−1^	1.04337 × 10^−1^
BinIPKO-TF10	Best	7.96648 × 10^5^	8.55467 × 10^5^	8.93782 × 10^5^	9.28942 × 10^5^
	Mean	7.99757 × 10^5^	8.57201 × 10^5^	8.94749 × 10^5^	9.29803 × 10^5^
	Std	1.21686 × 10^3^	9.05276 × 10^2^	9.38521 × 10^2^	1.66616 × 10^3^
	Gap	3.90234 × 10^−1^	2.92161 × 10^−1^	1.08125 × 10^−1^	9.27030 × 10^−2^
BinPKO-TF11	Best	8.09693 × 10^5^	8.73002 × 10^5^	9.25999 × 10^5^	9.86475 × 10^5^
	Mean	8.22828 × 10^5^	8.91012 × 10^5^	9.51383 × 10^5^	1.02698 × 10^6^
	Std	6.01281 × 10^3^	1.05434 × 10^4^	1.30880 × 10^4^	1.58659 × 10^4^
	Gap	3.28618 × 10^0^	4.24801 × 10^0^	6.44459 × 10^0^	1.05537 × 10^1^
BinIPKO-TF11	Best	8.05276 × 10^5^	8.73985 × 10^5^	9.12962 × 10^5^	9.78363 × 10^5^
	Mean	8.21513 × 10^5^	8.89765 × 10^5^	9.44013 × 10^5^	1.02261 × 10^6^
	Std	6.68806 × 10^3^	8.13190 × 10^3^	1.30560 × 10^4^	1.86474 × 10^4^
	Gap	3.12121 × 10^0^	4.10208 × 10^0^	5.61998 × 10^0^	1.00831 × 10^1^
BinPKO-TF12	Best	8.08918 × 10^5^	8.70586 × 10^5^	9.22297 × 10^5^	9.70917 × 10^5^
	Mean	8.22204 × 10^5^	8.88256 × 10^5^	9.45172 × 10^5^	1.02123 × 10^6^
	Std	6.64128 × 10^3^	9.66595 × 10^3^	1.19891 × 10^4^	2.04739 × 10^4^
	Gap	3.20783 × 10^0^	3.92556 × 10^0^	5.74972 × 10^0^	9.93530 × 10^0^
BinIPKO-TF12	Best	8.09627 × 10^5^	8.77943 × 10^5^	9.21250 × 10^5^	9.89585 × 10^5^
	Mean	8.22683 × 10^5^	8.91138 × 10^5^	9.44898 × 10^5^	1.02470 × 10^6^
	Std	7.33939 × 10^3^	5.97800 × 10^3^	1.25558 × 10^4^	1.64329 × 10^4^
	Gap	3.26798 × 10^0^	4.26270 × 10^0^	5.71910 × 10^0^	1.03080 × 10^1^
BinPKO-TF13	Best	8.10529 × 10^5^	8.79266 × 10^5^	9.13357 × 10^5^	9.87442 × 10^5^
	Mean	8.22917 × 10^5^	8.91150 × 10^5^	9.48391 × 10^5^	1.02785 × 10^6^
	Std	6.19321 × 10^3^	7.28259 × 10^3^	1.43674 × 10^4^	1.60481 × 10^4^
	Gap	3.29736 × 10^0^	4.26409 × 10^0^	6.10982 × 10^0^	1.06473 × 10^1^
BinIPKO-TF13	Best	8.05602 × 10^5^	8.70434 × 10^5^	9.37510 × 10^5^	9.77003 × 10^5^
	Mean	8.24208 × 10^5^	8.88578 × 10^5^	9.55590 × 10^5^	1.02142 × 10^6^
	Std	7.08162 × 10^3^	8.16380 × 10^3^	1.07977 × 10^4^	1.99370 × 10^4^
	Gap	3.45945 × 10^0^	3.96320 × 10^0^	6.91532 × 10^0^	9.95553 × 10^0^
BinPKO-TF14	Best	8.12815 × 10^5^	8.66163 × 10^5^	9.13113 × 10^5^	9.64350 × 10^5^
	Mean	8.24995 × 10^5^	8.87272 × 10^5^	9.41829 × 10^5^	1.02292 × 10^6^
	Std	5.21353 × 10^3^	1.12519 × 10^4^	1.28093 × 10^4^	1.81372 × 10^4^
	Gap	3.55818 × 10^0^	3.81044 × 10^0^	5.37574 × 10^0^	1.01162 × 10^1^
BinIPKO-TF14	Best	8.10178 × 10^5^	8.66168 × 10^5^	9.27941 × 10^5^	9.58855 × 10^5^
	Mean	8.24465 × 10^5^	8.89090 × 10^5^	9.45801 × 10^5^	1.01559 × 10^6^
	Std	6.87469 × 10^3^	8.30473 × 10^3^	1.12622 × 10^4^	2.13769 × 10^4^
	Gap	3.49166 × 10^0^	4.02316 × 10^0^	5.82009 × 10^0^	9.32805 × 10^0^

**Table 5:** Results obtained for Cap131, Cap132, Cap133, and Cap134 problems according to TFs.

TF	Criteria	Cap131	Cap132	Cap133	Cap134
BinPKO-TF1	Best	7.93440 × 10^5^	8.51495 × 10^5^	8.93077 × 10^5^	9.28942 × 10^5^
	Mean	7.95744 × 10^5^	8.53656 × 10^5^	8.94283 × 10^5^	9.29152 × 10^5^
	Std	1.83036 × 10^3^	2.48894 × 10^3^	9.05425 × 10^2^	5.00087 × 10^2^
	Gap	2.90477 × 10^−1^	2.53730 × 10^−1^	1.35041 × 10^−1^	2.26060 × 10^−2^
BinIPKO-TF1	Best	7.93440 × 10^5^	8.51495 × 10^5^	8.93077 × 10^5^	9.28942 × 10^5^
	Mean	7.93554 × 10^5^	8.51495 × 10^5^	8.93295 × 10^5^	9.28942 × 10^5^
	Std	2.97441 × 10^2^	5.92027 × 10^−10^	3.76595 × 10^2^	0.00000 × 10^0^
	Gap	1.44567 × 10^−2^	0.00000 × 10^0^	2.43914 × 10^−2^	0.00000 × 10^0^
BinPKO-TF2	Best	7.93440 × 10^5^	8.51495 × 10^5^	8.93077 × 10^5^	9.28942 × 10^5^
	Mean	7.98909 × 10^5^	8.56645 × 10^5^	8.95955 × 10^5^	9.29701 × 10^5^
	Std	4.03861 × 10^3^	3.09999 × 10^3^	2.83835 × 10^3^	1.95408 × 10^3^
	Gap	6.89355 × 10^−1^	6.04727 × 10^−1^	3.22293 × 10^−1^	8.17305 × 10^−2^
BinIPKO-TF2	Best	7.93440 × 10^5^	8.51495 × 10^5^	8.93077 × 10^5^	9.28942 × 10^5^
	Mean	7.94083 × 10^5^	8.51701 × 10^5^	8.93603 × 10^5^	9.28942 × 10^5^
	Std	1.00721 × 10^3^	5.07780 × 10^2^	5.32750 × 10^2^	0.00000 × 10^0^
	Gap	8.10563 × 10^−2^	2.41886 × 10^−2^	5.89222 × 10^−2^	0.00000 × 10^0^
BinPKO-TF3	Best	8.19741 × 10^5^	9.05389 × 10^5^	9.64104 × 10^5^	1.06948 × 10^6^
	Mean	8.29716 × 10^5^	9.15894 × 10^5^	9.88944 × 10^5^	1.09356 × 10^6^
	Std	3.77918 × 10^3^	5.88351 × 10^3^	1.05172 × 10^4^	1.34806 × 10^4^
	Gap	4.57200 × 10^0^	7.56303 × 10^0^	1.07345 × 10^1^	1.77213 × 10^1^
BinIPKO-TF3	Best	8.16417 × 10^5^	8.95754 × 10^5^	9.66357 × 10^5^	1.05666 × 10^6^
	Mean	8.30818 × 10^5^	9.15563 × 10^5^	9.90453 × 10^5^	1.09211 × 10^6^
	Std	4.08425 × 10^3^	8.44647 × 10^3^	1.08006 × 10^4^	1.92734 × 10^4^
	Gap	4.71093 × 10^0^	7.52411 × 10^0^	1.09035 × 10^1^	1.75650 × 10^1^
BinPKO-TF4	Best	8.21487 × 10^5^	9.01221 × 10^5^	9.76415 × 10^5^	1.06282 × 10^6^
	Mean	8.31690 × 10^5^	9.18139 × 10^5^	9.96565 × 10^5^	1.09727 × 10^6^
	Std	4.28930 × 10^3^	8.69661 × 10^3^	8.22245 × 10^3^	1.44981 × 10^4^
	Gap	4.82084 × 10^0^	7.82670 × 10^0^	1.15878 × 10^1^	1.81209 × 10^1^
BinIPKO-TF4	Best	8.23568 × 10^5^	9.02364 × 10^5^	9.60898 × 10^5^	1.03341 × 10^6^
	Mean	8.32745 × 10^5^	9.18302 × 10^5^	9.92484 × 10^5^	1.09439 × 10^6^
	Std	4.78583 × 10^3^	6.35166 × 10^3^	1.25641 × 10^4^	1.92490 × 10^4^
	Gap	4.95387 × 10^0^	7.84586 × 10^0^	1.11308 × 10^1^	1.78109 × 10^1^
BinPKO-TF5	Best	8.20085 × 10^5^	8.92917 × 10^5^	9.69358 × 10^5^	1.06112 × 10^6^
	Mean	8.30929 × 10^5^	9.14490 × 10^5^	9.85861 × 10^5^	1.08686 × 10^6^
	Std	4.58429 × 10^3^	7.46544 × 10^3^	9.06667 × 10^3^	1.34579 × 10^4^
	Gap	4.72489 × 10^0^	7.39809 × 10^0^	1.03893 × 10^1^	1.70000 × 10^1^
BinIPKO-TF5	Best	8.21467 × 10^5^	9.01631 × 10^5^	9.71180 × 10^5^	1.06534 × 10^6^
	Mean	8.31458 × 10^5^	9.14879 × 10^5^	9.88396 × 10^5^	1.08978 × 10^6^
	Std	3.54862 × 10^3^	6.25996 × 10^3^	7.76294 × 10^3^	1.48157 × 10^4^
	Gap	4.79165 × 10^0^	7.44383 × 10^0^	1.06731 × 10^1^	1.73141 × 10^1^
BinPKO-TF6	Best	8.19018 × 10^5^	8.96594 × 10^5^	9.69404 × 10^5^	1.05831 × 10^6^
	Mean	8.30942 × 10^5^	9.13843 × 10^5^	9.87061 × 10^5^	1.08524 × 10^6^
	Std	4.34832 × 10^3^	7.35362 × 10^3^	9.11592 × 10^3^	1.16862 × 10^4^
	Gap	4.72661 × 10^0^	7.32212 × 10^0^	1.05237 × 10^1^	1.68259 × 10^1^
BinIPKO-TF6	Best	8.24970 × 10^5^	9.02716 × 10^5^	9.54899 × 10^5^	1.03423 × 10^6^
	Mean	8.31678 × 10^5^	9.14710 × 10^5^	9.83919 × 10^5^	1.08494 × 10^6^
	Std	3.52286 × 10^3^	5.49625 × 10^3^	1.18609 × 10^4^	1.56446 × 10^4^
	Gap	4.81928 × 10^0^	7.42401 × 10^0^	1.01718 × 10^1^	1.67926 × 10^1^
BinPKO-TF7	Best	8.03370 × 10^5^	8.59952 × 10^5^	8.93252 × 10^5^	9.29478 × 10^5^
	Mean	8.11274 × 10^5^	8.72893 × 10^5^	9.15342 × 10^5^	9.65789 × 10^5^
	Std	3.73728 × 10^3^	6.18965 × 10^3^	1.10639 × 10^4^	1.74727 × 10^4^
	Gap	2.24776 × 10^0^	2.51297 × 10^0^	2.49315 × 10^0^	3.96656 × 10^0^
BinIPKO-TF7	Best	7.99106 × 10^5^	8.55187 × 10^5^	8.94664 × 10^5^	9.32592 × 10^5^
	Mean	8.08626 × 10^5^	8.67850 × 10^5^	9.09151 × 10^5^	9.52237 × 10^5^
	Std	4.05172 × 10^3^	5.16189 × 10^3^	7.37742 × 10^3^	1.06900 × 10^4^
	Gap	1.91400 × 10^0^	1.92069 × 10^0^	1.79983 × 10^0^	2.50774 × 10^0^
BinPKO-TF8	Best	8.04049 × 10^5^	8.56800 × 10^5^	9.00528 × 10^5^	9.30562 × 10^5^
	Mean	8.12307 × 10^5^	8.72111 × 10^5^	9.15252 × 10^5^	9.63548 × 10^5^
	Std	4.15541 × 10^3^	7.53774 × 10^3^	8.25412 × 10^3^	1.59497 × 10^4^
	Gap	2.37798 × 10^0^	2.42115 × 10^0^	2.48301 × 10^0^	3.72537 × 10^0^
BinIPKO-TF8	Best	8.06867 × 10^5^	8.54824 × 10^5^	9.00203 × 10^5^	9.28942 × 10^5^
	Mean	8.11039 × 10^5^	8.68105 × 10^5^	9.10941 × 10^5^	9.52179 × 10^5^
	Std	2.70513 × 10^3^	4.88606 × 10^3^	5.71475 × 10^3^	1.12558 × 10^4^
	Gap	2.21814 × 10^0^	1.95064 × 10^0^	2.00026 × 10^0^	2.50147 × 10^0^
BinPKO-TF9	Best	8.08349 × 10^5^	8.64715 × 10^5^	9.07755 × 10^5^	9.35123 × 10^5^
	Mean	8.14399 × 10^5^	8.75954 × 10^5^	9.20286 × 10^5^	9.64699 × 10^5^
	Std	3.40276 × 10^3^	5.16997 × 10^3^	6.56889 × 10^3^	1.35919 × 10^4^
	Gap	2.64163 × 10^0^	2.87248 × 10^0^	3.04671 × 10^0^	3.84925 × 10^0^
BinIPKO-TF9	Best	8.02290 × 10^5^	8.63723 × 10^5^	9.00843 × 10^5^	9.42784 × 10^5^
	Mean	8.12352 × 10^5^	8.71327 × 10^5^	9.14453 × 10^5^	9.62647 × 10^5^
	Std	4.04252 × 10^3^	3.54353 × 10^3^	6.08124 × 10^3^	1.28502 × 10^4^
	Gap	2.38357 × 10^0^	2.32904 × 10^0^	2.39352 × 10^0^	3.62832 × 10^0^
BinPKO-TF10	Best	8.10070 × 10^5^	8.68537 × 10^5^	9.03633 × 10^5^	9.44056 × 10^5^
	Mean	8.16355 × 10^5^	8.76007 × 10^5^	9.17637 × 10^5^	9.67127 × 10^5^
	Std	3.25408 × 10^3^	3.89242 × 10^3^	5.45872 × 10^3^	1.02862 × 10^4^
	Gap	2.88806 × 10^0^	2.87868 × 10^0^	2.75002 × 10^0^	4.11066 × 10^0^
BinIPKO-TF10	Best	8.08092 × 10^5^	8.66000 × 10^5^	9.04728 × 10^5^	9.47674 × 10^5^
	Mean	8.14749 × 10^5^	8.74959 × 10^5^	9.17259 × 10^5^	9.65874 × 10^5^
	Std	3.35725 × 10^3^	3.78776 × 10^3^	5.88845 × 10^3^	8.65389 × 10^3^
	Gap	2.68572 × 10^0^	2.75554 × 10^0^	2.70770 × 10^0^	3.97577 × 10^0^
BinPKO-TF11	Best	8.48670 × 10^5^	9.48259 × 10^5^	1.03814 × 10^6^	1.17130 × 10^6^
	Mean	8.75153 × 10^5^	9.94077 × 10^5^	1.10123 × 10^6^	1.24969 × 10^6^
	Std	1.15472 × 10^4^	1.86790 × 10^4^	2.55738 × 10^4^	3.81273 × 10^4^
	Gap	1.02986 × 10^1^	1.67448 × 10^1^	2.33078 × 10^1^	3.45282 × 10^1^
BinIPKO-TF11	Best	8.60426 × 10^5^	9.62452 × 10^5^	1.04368 × 10^6^	1.20114 × 10^6^
	Mean	8.75531 × 10^5^	9.96982 × 10^5^	1.10822 × 10^6^	1.26096 × 10^6^
	Std	9.90571 × 10^3^	1.37942 × 10^4^	2.68366 × 10^4^	3.06623 × 10^4^
	Gap	1.03463 × 10^1^	1.70860 × 10^1^	2.40897 × 10^1^	3.57411 × 10^1^
BinPKO-TF12	Best	8.48973 × 10^5^	9.64051 × 10^5^	1.03484 × 10^6^	1.14920 × 10^6^
	Mean	8.73764 × 10^5^	9.87757 × 10^5^	1.08623 × 10^6^	1.22907 × 10^6^
	Std	9.28738 × 10^3^	1.30846 × 10^4^	2.17634 × 10^4^	4.05566 × 10^4^
	Gap	1.01236 × 10^1^	1.60026 × 10^1^	2.16281 × 10^1^	3.23084 × 10^1^
BinIPKO-TF12	Best	8.55156 × 10^5^	9.59776 × 10^5^	1.00755 × 10^6^	1.15256 × 10^6^
	Mean	8.71691 × 10^5^	9.81465 × 10^5^	1.08979 × 10^6^	1.23707 × 10^6^
	Std	1.01954 × 10^4^	1.27011 × 10^4^	2.82388 × 10^4^	3.34929 × 10^4^
	Gap	9.86227 × 10^0^	1.52637 × 10^1^	2.20261 × 10^1^	3.31696 × 10^1^
BinPKO-TF13	Best	8.51444 × 10^5^	9.55657 × 10^5^	1.01875 × 10^6^	1.16489 × 10^6^
	Mean	8.73039 × 10^5^	9.84412 × 10^5^	1.08140 × 10^6^	1.23800 × 10^6^
	Std	8.67566 × 10^3^	1.32521 × 10^4^	2.46270 × 10^4^	2.84772 × 10^4^
	Gap	1.00322 × 10^1^	1.56098 × 10^1^	2.10866 × 10^1^	3.32700 × 10^1^
BinIPKO-TF13	Best	8.47396 × 10^5^	9.48811 × 10^5^	1.04075 × 10^6^	1.13664 × 10^6^
	Mean	8.70010 × 10^5^	9.84343 × 10^5^	1.08763 × 10^6^	1.23016 × 10^6^
	Std	9.91108 × 10^3^	1.48656 × 10^4^	2.51769 × 10^4^	4.02794 × 10^4^
	Gap	9.65043 × 10^0^	1.56016 × 10^1^	2.17842 × 10^1^	3.24264 × 10^1^
BinPKO-TF14	Best	8.56100 × 10^5^	9.51041 × 10^5^	1.03463 × 10^6^	1.14607 × 10^6^
	Mean	8.73557 × 10^5^	9.78055 × 10^5^	1.08099 × 10^6^	1.22025 × 10^6^
	Std	1.05721 × 10^4^	1.50596 × 10^4^	1.95972 × 10^4^	3.03883 × 10^4^
	Gap	1.00975 × 10^1^	1.48633 × 10^1^	2.10416 × 10^1^	3.13592 × 10^1^
BinIPKO-TF14	Best	8.54288 × 10^5^	9.45346 × 10^5^	1.02589 × 10^6^	1.13448 × 10^6^
	Mean	8.69417 × 10^5^	9.80105 × 10^5^	1.08315 × 10^6^	1.22875 × 10^6^
	Std	9.04685 × 10^3^	1.73450 × 10^4^	2.37115 × 10^4^	3.10264 × 10^4^
	Gap	9.57565 × 10^0^	1.51040 × 10^1^	2.12825 × 10^1^	3.22740 × 10^1^

**Table 6:** Results obtained for CapA, CapB, and CapC problems according to TFs.

TF	Criteria	CapA	CapB	CapC
BinPKO-TF1	Best	1.71565 × 10^7^	1.30191 × 10^7^	1.15308 × 10^7^
	Mean	1.77950 × 10^7^	1.33178 × 10^7^	1.18429 × 10^7^
	Std	4.42841 × 10^5^	1.71254 × 10^5^	1.34772 × 10^5^
	Gap	3.72188 × 10^0^	2.60956 × 10^0^	2.93190 × 10^0^
BinIPKO-TF1	Best	1.71565 × 10^7^	1.29791 × 10^7^	1.15094 × 10^7^
	Mean	1.71995 × 10^7^	1.30691 × 10^7^	1.16009 × 10^7^
	Std	8.06209 × 10^4^	5.25145 × 10^4^	5.48158 × 10^4^
	Gap	2.51154 × 10^−1^	6.93514 × 10^−1^	8.28749 × 10^−1^
BinPKO-TF2	Best	1.71565 × 10^7^	1.29791 × 10^7^	1.16782 × 10^7^
	Mean	1.78138 × 10^7^	1.34044 × 10^7^	1.19615 × 10^7^
	Std	4.48612 × 10^5^	2.28607 × 10^5^	1.87604 × 10^5^
	Gap	3.83142 × 10^0^	3.27709 × 10^0^	3.96234 × 10^0^
BinIPKO-TF2	Best	1.71565 × 10^7^	1.30573 × 10^7^	1.15446 × 10^7^
	Mean	1.78233 × 10^7^	1.33945 × 10^7^	1.17920 × 10^7^
	Std	6.27078 × 10^5^	2.88737 × 10^5^	1.43806 × 10^5^
	Gap	3.88672 × 10^0^	3.20093 × 10^0^	2.48965 × 10^0^
BinPKO-TF3	Best	5.71948 × 10^7^	2.75484 × 10^7^	2.10996 × 10^7^
	Mean	6.33999 × 10^7^	2.94208 × 10^7^	2.23612 × 10^7^
	Std	2.45707 × 10^6^	9.64487 × 10^5^	5.85699 × 10^5^
	Gap	2.69540 × 10^2^	1.26679 × 10^2^	9.43505 × 10^1^
BinIPKO-TF3	Best	5.70545 × 10^7^	2.75217 × 10^7^	2.16105 × 10^7^
	Mean	6.30430 × 10^7^	2.91523 × 10^7^	2.26801 × 10^7^
	Std	2.81273 × 10^6^	8.63278 × 10^5^	5.81432 × 10^5^
	Gap	2.67460 × 10^2^	1.24610 × 10^2^	9.71222 × 10^1^
BinPKO-TF4	Best	5.90987 × 10^7^	2.70574 × 10^7^	2.01927 × 10^7^
	Mean	6.30919 × 10^7^	2.91384 × 10^7^	2.19720 × 10^7^
	Std	2.00824 × 10^6^	8.86741 × 10^5^	7.56766 × 10^5^
	Gap	2.67745 × 10^2^	1.24503 × 10^2^	9.09684 × 10^1^
BinIPKO-TF4	Best	5.78289 × 10^7^	2.63409 × 10^7^	2.02061 × 10^7^
	Mean	6.30251 × 10^7^	2.89073 × 10^7^	2.19114 × 10^7^
	Std	2.16834 × 10^6^	8.99640 × 10^5^	6.76432 × 10^5^
	Gap	2.67355 × 10^2^	1.22723 × 10^2^	9.04413 × 10^1^
BinPKO-TF5	Best	5.26343 × 10^7^	2.39854 × 10^7^	2.07397 × 10^7^
	Mean	6.07820 × 10^7^	2.81698 × 10^7^	2.16994 × 10^7^
	Std	2.91145 × 10^6^	1.20191 × 10^6^	4.57414 × 10^5^
	Gap	2.54281 × 10^2^	1.17040 × 10^2^	8.85985 × 10^1^
BinIPKO-TF5	Best	5.27869 × 10^7^	2.72044 × 10^7^	2.02129 × 10^7^
	Mean	6.11866 × 10^7^	2.85107 × 10^7^	2.14842 × 10^7^
	Std	2.34815 × 10^6^	7.17351 × 10^5^	6.04955 × 10^5^
	Gap	2.56639 × 10^2^	1.19666 × 10^2^	8.67283 × 10^1^
BinPKO-TF6	Best	5.53381 × 10^7^	2.58254 × 10^7^	2.00351 × 10^7^
	Mean	6.00632 × 10^7^	2.75460 × 10^7^	2.12791 × 10^7^
	Std	2.13342 × 10^6^	8.18270 × 10^5^	6.04248 × 10^5^
	Gap	2.50091 × 10^2^	1.12234 × 10^2^	8.49458 × 10^1^
BinIPKO-TF6	Best	5.59181 × 10^7^	2.48964 × 10^7^	2.04015 × 10^7^
	Mean	6.01487 × 10^7^	2.78610 × 10^7^	2.12888 × 10^7^
	Std	1.93780 × 10^6^	1.19760 × 10^6^	4.33060 × 10^5^
	Gap	2.50590 × 10^2^	1.14661 × 10^2^	8.50296 × 10^1^
BinPKO-TF7	Best	2.03764 × 10^7^	1.57416 × 10^7^	1.32316 × 10^7^
	Mean	3.36916 × 10^7^	1.82940 × 10^7^	1.47831 × 10^7^
	Std	4.00894 × 10^6^	1.17071 × 10^6^	7.00122 × 10^5^
	Gap	9.63785 × 10^1^	4.09503 × 10^1^	2.84861 × 10^1^
BinIPKO-TF7	Best	2.38745 × 10^7^	1.44706 × 10^7^	1.33923 × 10^7^
	Mean	2.93452 × 10^7^	1.67204 × 10^7^	1.42944 × 10^7^
	Std	2.44130 × 10^6^	1.03206 × 10^6^	5.09938 × 10^5^
	Gap	7.10449 × 10^1^	2.88262 × 10^1^	2.42385 × 10^1^
BinPKO-TF8	Best	2.65171 × 10^7^	1.60146 × 10^7^	1.30241 × 10^7^
	Mean	3.22467 × 10^7^	1.79422 × 10^7^	1.48652 × 10^7^
	Std	3.12743 × 10^6^	8.26445 × 10^5^	6.12360 × 10^5^
	Gap	8.79567 × 10^1^	3.82391 × 10^1^	2.91998 × 10^1^
BinIPKO-TF8	Best	2.41268 × 10^7^	1.52273 × 10^7^	1.34486 × 10^7^
	Mean	3.01530 × 10^7^	1.69125 × 10^7^	1.42031 × 10^7^
	Std	2.37732 × 10^6^	8.25919 × 10^5^	3.95200 × 10^5^
	Gap	7.57529 × 10^1^	3.03062 × 10^1^	2.34455 × 10^1^
BinPKO-TF9	Best	2.66924 × 10^7^	1.49497 × 10^7^	1.32327 × 10^7^
	Mean	3.16059 × 10^7^	1.75518 × 10^7^	1.44936 × 10^7^
	Std	2.23648 × 10^6^	9.49793 × 10^5^	4.99413 × 10^5^
	Gap	8.42218 × 10^1^	3.52317 × 10^1^	2.59696 × 10^1^
BinIPKO-TF9	Best	2.66589 × 10^7^	1.56835 × 10^7^	1.31494 × 10^7^
	Mean	3.07329 × 10^7^	1.71333 × 10^7^	1.42215 × 10^7^
	Std	1.74879 × 10^6^	6.90253 × 10^5^	4.09186 × 10^5^
	Gap	7.91335 × 10^1^	3.20075 × 10^1^	2.36050 × 10^1^
BinPKO-TF10	Best	2.66908 × 10^7^	1.59514 × 10^7^	1.32660 × 10^7^
	Mean	3.01545 × 10^7^	1.70191 × 10^7^	1.43033 × 10^7^
	Std	1.54591 × 10^6^	5.25766 × 10^5^	3.92094 × 10^5^
	Gap	7.57616 × 10^1^	3.11273 × 10^1^	2.43159 × 10^1^
BinIPKO-TF10	Best	2.46667 × 10^7^	1.54393 × 10^7^	1.31880 × 10^7^
	Mean	2.93951 × 10^7^	1.68527 × 10^7^	1.41018 × 10^7^
	Std	1.95873 × 10^6^	6.04234 × 10^5^	3.61209 × 10^5^
	Gap	7.13356 × 10^1^	2.98454 × 10^1^	2.25649 × 10^1^
BinPKO-TF11	Best	7.50320 × 10^7^	3.40871 × 10^7^	2.33666 × 10^7^
	Mean	8.50539 × 10^7^	3.74677 × 10^7^	2.78992 × 10^7^
	Std	5.18131 × 10^6^	1.43298 × 10^6^	1.41356 × 10^6^
	Gap	3.95755 × 10^2^	1.88678 × 10^2^	1.42484 × 10^2^
BinIPKO-TF11	Best	7.15677 × 10^7^	3.01865 × 10^7^	2.58839 × 10^7^
	Mean	8.49941 × 10^7^	3.73931 × 10^7^	2.80055 × 10^7^
	Std	5.30995 × 10^6^	2.04611 × 10^6^	1.15705 × 10^6^
	Gap	3.95406 × 10^2^	1.88103 × 10^2^	1.43408 × 10^2^
BinPKO-TF12	Best	6.94863 × 10^7^	3.15054 × 10^7^	2.36680 × 10^7^
	Mean	7.86782 × 10^7^	3.54019 × 10^7^	2.65078 × 10^7^
	Std	4.19309 × 10^6^	1.75698 × 10^6^	1.28555 × 10^6^
	Gap	3.58592 × 10^2^	1.72761 × 10^2^	1.30390 × 10^2^
BinIPKO-TF12	Best	6.96565 × 10^7^	3.26295 × 10^7^	2.35454 × 10^7^
	Mean	7.94519 × 10^7^	3.55172 × 10^7^	2.68609 × 10^7^
	Std	4.31017 × 10^6^	1.39825 × 10^6^	1.23503 × 10^6^
	Gap	3.63102 × 10^2^	1.73650 × 10^2^	1.33460 × 10^2^
BinPKO-TF13	Best	6.53079 × 10^7^	3.24269 × 10^7^	2.45539 × 10^7^
	Mean	7.83272 × 10^7^	3.55511 × 10^7^	2.62887 × 10^7^
	Std	4.64353 × 10^6^	1.45012 × 10^6^	9.75295 × 10^5^
	Gap	3.56547 × 10^2^	1.73911 × 10^2^	1.28486 × 10^2^
BinIPKO-TF13	Best	6.90070 × 10^7^	3.16039 × 10^7^	2.49922 × 10^7^
	Mean	7.84538 × 10^7^	3.52163 × 10^7^	2.65232 × 10^7^
	Std	3.92122 × 10^6^	1.62699 × 10^6^	7.91992 × 10^5^
	Gap	3.57285 × 10^2^	1.71331 × 10^2^	1.30524 × 10^2^
BinPKO-TF14	Best	6.98460 × 10^7^	3.17333 × 10^7^	2.26788 × 10^7^
	Mean	7.84517 × 10^7^	3.43663 × 10^7^	2.60094 × 10^7^
	Std	3.59008 × 10^6^	1.31364 × 10^6^	1.44352 × 10^6^
	Gap	3.57272 × 10^2^	1.64782 × 10^2^	1.26059 × 10^2^
BinIPKO-TF14	Best	7.13471 × 10^7^	3.07604 × 10^7^	2.41374 × 10^7^
	Mean	7.80793 × 10^7^	3.48597 × 10^7^	2.62398 × 10^7^
	Std	3.97784 × 10^6^	1.80289 × 10^6^	1.02363 × 10^6^
	Gap	3.55101 × 10^2^	1.68584 × 10^2^	1.28061 × 10^2^

**Table 7:** Ranking of best values obtained according to TFs.

Problems	TF1	TF2	TF3	TF4	TF5	TF6	TF7	TF8	TF9	TF10	TF11	TF12	TF13	TF14
Cap71	1	1	1	1	1	1	1	1	1	1	3	5	4	2
Cap72	1	1	1	1	1	1	1	1	1	1	3	5	2	4
Cap73	1	1	1	1	1	1	1	1	1	1	5	3	2	4
Cap74	1	1	1	1	1	1	1	1	1	1	3	2	4	5
Cap101	1	1	1	2	3	4	1	1	1	1	5	7	6	8
Cap102	1	1	3	4	5	6	1	1	1	2	9	10	8	7
Cap103	1	1	2	3	4	5	1	1	1	1	6	7	9	8
Cap104	1	1	2	3	2	3	1	1	1	1	6	7	5	4
Cap131	1	1	6	8	7	9	2	4	3	5	13	12	10	11
Cap132	1	1	6	8	7	9	3	2	4	5	13	12	11	10
Cap133	1	1	8	7	9	6	2	3	4	5	13	10	12	11
Cap134	1	1	7	5	8	6	2	1	3	4	12	11	10	9
CapA	1	1	8	9	6	7	2	3	5	4	13	11	10	12
CapB	1	2	10	8	9	7	3	4	6	5	11	14	13	12
CapC	1	2	10	7	8	9	5	6	3	4	14	11	13	12
Mean Rank	1.00	1.13	4.47	4.53	4.80	5.00	1.80	2.07	2.40	2.73	8.60	8.47	7.93	7.93
Final Rank	1	2	7	8	9	10	3	4	5	6	13	14	12	11

**Table 8:** Ranking of mean values obtained according to TFs.

Problems	TF1	TF2	TF3	TF4	TF5	TF6	TF7	TF8	TF9	TF10	TF11	TF12	TF13	TF14
Cap71	1	1	1	1	2	1	1	1	1	1	3	6	5	4
Cap72	1	1	1	1	2	2	1	1	1	1	3	6	4	5
Cap73	1	1	2	3	5	4	1	1	1	1	8	9	7	6
Cap74	1	1	1	3	4	2	1	1	1	1	5	7	6	8
Cap101	2	1	5	7	9	10	3	4	6	8	11	12	13	14
Cap102	1	1	6	7	8	9	2	3	4	5	12	13	10	11
Cap103	1	2	7	8	10	9	3	5	4	6	11	12	14	13
Cap104	1	1	5	8	7	6	2	3	2	4	11	12	10	9
Cap131	1	2	7	10	8	9	3	4	5	6	14	13	12	11
Cap132	1	2	9	10	8	7	3	4	5	6	14	12	13	11
Cap133	1	2	9	10	8	7	3	4	5	6	14	13	12	11
Cap134	1	1	8	9	7	6	3	2	4	5	13	12	11	10
CapA	1	2	10	9	8	7	3	5	6	4	14	13	12	11
CapB	1	2	10	9	8	7	3	5	6	4	14	13	12	11
CapC	1	2	10	9	8	7	6	4	5	3	14	13	12	11
Mean Rank	1.07	1.47	6.07	6.93	6.80	6.20	2.53	3.13	3.73	4.07	10.73	11.07	10.20	9.73
Final Rank	1	2	7	8	9	10	3	6	5	4	14	13	11	12

**Table 9:** Results obtained by the algorithms according to problems.

		GWO	APO	PSO	EEFO	BinPKO	BinIPKO
Cap71	Best	9.32616 × 10^5^	9.32616 × 10^5^	9.50470 × 10^5^	9.32616 × 10^5^	9.32616 × 10^5^	9.32616 × 10^5^
	Mean	9.32908 × 10^5^	9.34126 × 10^5^	9.50470 × 10^5^	9.32616 × 10^5^	9.32616 × 10^5^	9.32616 × 10^5^
	Std	5.88039 × 10^2^	1.12346 × 10^3^	0.00000 × 10^0^	0.00000 × 10^0^	0.00000 × 10^0^	0.00000 × 10^0^
	Gap	3.13791 × 10^−2^	1.61989 × 10^−1^	1.91445 × 10^0^	0.00000 × 10^0^	0.00000 × 10^0^	0.00000 × 10^0^
Cap72	Best	9.77799 × 10^5^	9.77799 × 10^5^	1.02547 × 10^6^	9.77799 × 10^5^	9.77799 × 10^5^	9.77799 × 10^5^
	Mean	9.79050 × 10^5^	9.78496 × 10^5^	1.02547 × 10^6^	9.77799 × 10^5^	9.77799 × 10^5^	9.77799 × 10^5^
	Std	1.37031 × 10^3^	7.60154 × 10^2^	0.00000 × 10^0^	4.73622 × 10^−10^	4.73622 × 10^−10^	4.73622 × 10^−10^
	Gap	1.27914 × 10^−1^	7.12151 × 10^−2^	4.87531 × 10^0^	0.00000 × 10^0^	0.00000 × 10^0^	0.00000 × 10^0^
Cap73	Best	1.01064 × 10^6^	1.01064 × 10^6^	1.10047 × 10^6^	1.01064 × 10^6^	1.01064 × 10^6^	1.01064 × 10^6^
	Mean	1.01071 × 10^6^	1.01067 × 10^6^	1.10047 × 10^6^	1.01080 × 10^6^	1.01064 × 10^6^	1.01064 × 10^6^
	Std	3.35689 × 10^2^	6.31922 × 10^1^	2.36811 × 10^−10^	3.45135 × 10^2^	4.73622 × 10^−10^	4.73622 × 10^−10^
	Gap	7.15371 × 10^−3^	2.74929 × 10^−3^	8.88829 × 10^0^	1.52198 × 10^−2^	0.00000 × 10^0^	0.00000 × 10^0^
Cap74	Best	1.03498 × 10^6^	1.03498 × 10^6^	1.21297 × 10^6^	1.03498 × 10^6^	1.03498 × 10^6^	1.03498 × 10^6^
	Mean	1.03516 × 10^6^	1.03516 × 10^6^	1.21297 × 10^6^	1.03631 × 10^6^	1.03498 × 10^6^	1.03498 × 10^6^
	Std	6.95186 × 10^2^	6.95186 × 10^2^	0.00000 × 10^0^	1.61778 × 10^3^	3.55216 × 10^−10^	3.55216 × 10^−10^
	Gap	1.76500 × 10^−2^	1.76500 × 10^−2^	1.71978 × 10^1^	1.29082 × 10^−1^	0.00000 × 10^0^	0.00000 × 10^0^
Cap101	Best	7.97602 × 10^5^	8.00005 × 10^5^	8.32291 × 10^5^	7.97509 × 10^5^	7.96648 × 10^5^	7.96648 × 10^5^
	Mean	8.01985 × 10^5^	8.03968 × 10^5^	8.32291 × 10^5^	7.98637 × 10^5^	7.96763 × 10^5^	7.96677 × 10^5^
	Std	2.06807 × 10^3^	1.86444 × 10^3^	3.55216 × 10^−10^	9.11208 × 10^2^	2.97441 × 10^2^	1.57066 × 10^2^
	Gap	6.69935 × 10^−1^	9.18847 × 10^−1^	4.47408 × 10^0^	2.49678 × 10^−1^	1.43984 × 10^−2^	3.59961 × 10^−3^
Cap102	Best	8.54704 × 10^5^	8.56734 × 10^5^	9.52291 × 10^5^	8.55781 × 10^5^	8.54704 × 10^5^	8.54704 × 10^5^
	Mean	8.59881 × 10^5^	8.61241 × 10^5^	9.52291 × 10^5^	8.60039 × 10^5^	8.54782 × 10^5^	8.54704 × 10^5^
	Std	3.28770 × 10^3^	1.96550 × 10^3^	5.92027 × 10^−10^	2.30635 × 10^3^	2.98455 × 10^2^	5.92027 × 10^−10^
	Gap	6.05675 × 10^−1^	7.64837 × 10^−1^	1.14176 × 10^1^	6.24177 × 10^−1^	9.14332 × 10^−3^	0.00000 × 10^0^
Cap103	Best	8.94008 × 10^5^	8.94008 × 10^5^	1.07229 × 10^6^	8.97708 × 10^5^	8.93782 × 10^5^	8.93782 × 10^5^
	Mean	8.98411 × 10^5^	8.98643 × 10^5^	1.07229 × 10^6^	9.03518 × 10^5^	8.94035 × 10^5^	8.93790 × 10^5^
	Std	3.79111 × 10^3^	2.59175 × 10^3^	7.10433 × 10^−10^	3.23733 × 10^3^	4.42600 × 10^2^	4.12663 × 10^1^
	Gap	5.17867 × 10^−1^	5.43829 × 10^−1^	1.99723 × 10^1^	1.08934 × 10^0^	2.82751 × 10^−2^	8.42953 × 10^−4^
Cap104	Best	9.28942 × 10^5^	9.28942 × 10^5^	1.25229 × 10^6^	9.40734 × 10^5^	9.28942 × 10^5^	9.28942 × 10^5^
	Mean	9.32338 × 10^5^	9.37800 × 10^5^	1.25229 × 10^6^	9.50757 × 10^5^	9.28942 × 10^5^	9.28942 × 10^5^
	Std	4.49298 × 10^3^	4.80656 × 10^3^	9.47244 × 10^−10^	5.33539 × 10^3^	0.00000 × 10^0^	0.00000 × 10^0^
	Gap	3.65615 × 10^−1^	9.53598 × 10^−1^	3.48084 × 10^1^	2.34837 × 10^0^	0.00000 × 10^0^	0.00000 × 10^0^
Cap131	Best	8.09469 × 10^5^	8.18840 × 10^5^	9.91571 × 10^5^	8.19739 × 10^5^	7.93440 × 10^5^	7.93440 × 10^5^
	Mean	8.20570 × 10^5^	8.24930 × 10^5^	9.91571 × 10^5^	8.31008 × 10^5^	7.95744 × 10^5^	7.93554 × 10^5^
	Std	5.85498 × 10^3^	2.79470 × 10^3^	4.73622 × 10^−10^	4.24606 × 10^3^	1.83036 × 10^3^	2.97441 × 10^2^
	Gap	3.41929 × 10^0^	3.96885 × 10^0^	2.49713 × 10^1^	4.73494 × 10^0^	2.90477 × 10^−1^	1.44567 × 10^−2^
Cap132	Best	8.58255 × 10^5^	8.81210 × 10^5^	1.23657 × 10^6^	8.86161 × 10^5^	8.51495 × 10^5^	8.51495 × 10^5^
	Mean	8.78467 × 10^5^	8.93002 × 10^5^	1.23657 × 10^6^	9.12404 × 10^5^	8.53656 × 10^5^	8.51495 × 10^5^
	Std	8.85584 × 10^3^	4.82812 × 10^3^	2.36811 × 10^−10^	6.86313 × 10^3^	2.48894 × 10^3^	5.92027 × 10^−10^
	Gap	3.16752 × 10^0^	4.87460 × 10^0^	4.52235 × 10^1^	7.15310 × 10^0^	2.53730 × 10^−1^	0.00000 × 10^0^
Cap133	Best	8.96749 × 10^5^	9.14777 × 10^5^	1.48157 × 10^6^	9.66744 × 10^5^	8.93077 × 10^5^	8.93077 × 10^5^
	Mean	9.15475 × 10^5^	9.41639 × 10^5^	1.48157 × 10^6^	9.86021 × 10^5^	8.94283 × 10^5^	8.93295 × 10^5^
	Std	1.32593 × 10^4^	8.82168 × 10^3^	4.73622 × 10^−10^	8.58664 × 10^3^	9.05425 × 10^2^	3.76595 × 10^2^
	Gap	2.50804 × 10^0^	5.43766 × 10^0^	6.58952 × 10^1^	1.04072 × 10^1^	1.35041 × 10^−1^	2.43914 × 10^−2^
Cap134	Best	9.28942 × 10^5^	9.93181 × 10^5^	1.84907 × 10^6^	1.03046 × 10^6^	9.28942 × 10^5^	9.28942 × 10^5^
	Mean	9.53368 × 10^5^	1.01412 × 10^6^	1.84907 × 10^6^	1.07313 × 10^6^	9.29152 × 10^5^	9.28942 × 10^5^
	Std	2.30917 × 10^4^	1.17946 × 10^4^	1.18405 × 10^−9^	1.70366 × 10^4^	5.00087 × 10^2^	0.00000 × 10^0^
	Gap	2.62948 × 10^0^	9.16924 × 10^0^	9.90514 × 10^1^	1.55216 × 10^1^	2.26060 × 10^−2^	0.00000 × 10^0^
CapA	Best	1.73468 × 10^7^	3.65634 × 10^7^	1.82644 × 10^8^	5.36399 × 10^7^	1.71565 × 10^7^	1.71565 × 10^7^
	Mean	1.80642 × 10^7^	4.09759 × 10^7^	1.82644 × 10^8^	5.73725 × 10^7^	1.77950 × 10^7^	1.71995 × 10^7^
	Std	4.07503 × 10^5^	1.96277 × 10^6^	0.00000 × 10^0^	2.05592 × 10^6^	4.42841 × 10^5^	8.06209 × 10^4^
	Gap	5.29117 × 10^0^	1.38836 × 10^2^	9.64576 × 10^2^	2.34407 × 10^2^	3.72188 × 10^0^	2.51154 × 10^−1^
CapB	Best	1.35195 × 10^7^	1.96494 × 10^7^	7.66358 × 10^7^	2.53495 × 10^7^	1.30191 × 10^7^	1.29791 × 10^7^
	Mean	1.38501 × 10^7^	2.08074 × 10^7^	7.66358 × 10^7^	2.68215 × 10^7^	1.33178 × 10^7^	1.30691 × 10^7^
	Std	1.31085 × 10^5^	6.04358 × 10^5^	4.54677 × 10^−8^	7.97178 × 10^5^	1.71254 × 10^5^	5.25145 × 10^4^
	Gap	6.71116 × 10^0^	6.03149 × 10^1^	4.90456 × 10^2^	1.06652 × 10^2^	2.60956 × 10^0^	6.93514 × 10^−1^
CapC	Best	1.19246 × 10^7^	1.52086 × 10^7^	5.59415 × 10^7^	1.90084 × 10^7^	1.15308 × 10^7^	1.15094 × 10^7^
	Mean	1.22613 × 10^7^	1.65162 × 10^7^	5.59415 × 10^7^	2.07528 × 10^7^	1.18429 × 10^7^	1.16009 × 10^7^
	Std	1.55385 × 10^5^	5.25349 × 10^5^	1.51559 × 10^−8^	5.51998 × 10^5^	1.34772 × 10^5^	5.48158 × 10^4^
	Gap	6.56846 × 10^0^	4.35494 × 10^1^	3.86211 × 10^2^	8.03717 × 10^1^	2.93190 × 10^0^	8.28749 × 10^−1^
TOPSIS	Value	5.48 × 10^−2^	5.91 × 10^−3^	0.00 × 10^0^	3.09 × 10^−3^	1.81 × 10^−1^	1.00 × 10^0^
	Rank	3	4	6	5	2	1
PROMETHEE	Value	3.00 × 10^0^	2.00 × 10^0^	0.00 × 10^0^	1.00 × 10^0^	4.00 × 10^0^	5.00 × 10^0^
	Rank	3	4	6	5	2	1
Friedman mean ranks		3.4	4.1	6.0	4.4	1.9	1.2
Rank		3	4	6	5	2	1
*p*-value		5.7847 × 10^−13^					

**Table 10:** Ranking of the mean values obtained by the algorithms.

Problems	GWO	APO	PSO	EEFO	BinPKO	BinIPKO
Cap71	2	3	4	1	1	1
Cap72	3	2	4	1	1	1
Cap73	3	2	5	4	1	1
Cap74	2	2	4	3	1	1
Cap101	4	5	6	3	2	1
Cap102	3	5	6	4	2	1
Cap103	3	4	6	5	2	1
Cap104	2	3	5	4	1	1
Cap131	3	4	6	5	2	1
Cap132	3	4	6	5	2	1
Cap133	3	4	6	5	2	1
Cap134	3	4	6	5	2	1
CapA	3	4	6	5	2	1
CapB	3	4	6	5	2	1
CapC	3	4	6	5	2	1
Mean Rank	2.87	3.60	5.47	4.00	1.67	1.00
Final Rank	3	4	6	5	2	1

## Data Availability

No new data were created or analyzed in this study.
